# Geochemical transition zone powering microbial growth in subsurface sediments

**DOI:** 10.1073/pnas.2005917117

**Published:** 2020-12-07

**Authors:** Rui Zhao, José M. Mogollón, Sophie S. Abby, Christa Schleper, Jennifer F. Biddle, Desiree L. Roerdink, Ingunn H. Thorseth, Steffen L. Jørgensen

**Affiliations:** ^a^K.G. Jebsen Centre for Deep Sea Research, University of Bergen, 5007 Bergen, Norway;; ^b^School of Marine Science and Policy, University of Delaware, Lewes, DE 19958;; ^c^Institute of Environmental Sciences (CML), Leiden University, 2333 CC Leiden, The Netherlands;; ^d^Division of Archaea Biology and Ecogenomics, Department of Functional and Evolutionary Ecology, University of Vienna, A-1090 Vienna, Austria

**Keywords:** deep biosphere, microbial in situ growth, nitrogen cycle, energy availability, anammox

## Abstract

No other environment hosts as many microbial cells as the marine sedimentary biosphere. While the majority of these cells are expected to be alive, they are speculated to be persisting in a state of maintenance without net growth due to extreme starvation. Here, we report evidence for in situ growth of anaerobic ammonium-oxidizing (anammox) bacteria in ∼80,000-y-old subsurface sediments from the Arctic Mid-Ocean Ridge. The growth is confined to the nitrate–ammonium transition zone (NATZ), a widespread geochemical transition zone where most of the upward ammonium flux from deep anoxic sediments is being consumed. In this zone the anammox bacteria abundances, assessed by quantification of marker genes, consistently displayed a four order of magnitude increase relative to adjacent layers in four cores. This subsurface cell increase coincides with a markedly higher power supply driven mainly by intensified anammox reaction rates, thereby providing a quantitative link between microbial proliferation and energy availability. The reconstructed draft genome of the dominant anammox bacterium showed an index of replication (iRep) of 1.32, suggesting that 32% of this population was actively replicating. The genome belongs to a *Scalindua* species which we name *Candidatus Scalindua sediminis*, so far exclusively found in marine sediments. It has the capacity to utilize urea and cyanate and a mixotrophic lifestyle. Our results demonstrate that specific microbial groups are not only able to survive unfavorable conditions over geological timescales, but can proliferate in situ when encountering ideal conditions with significant consequences for biogeochemical nitrogen cycling.

## Significance

The marine sedimentary subsurface is a vast and inhospitable ecosystem, often described as a place where microbes “race to their death,” as microbial cells are buried and available energy is severely diminished with increasing depth/age. By combining a variety of biogeochemical and molecular methods to describe the energetics and genetics of the bacteria specialized in anaerobic ammonium oxidation, we show that despite prolonged exposure to highly unfavorable conditions for tens of thousands of years, these bacteria exhibit remarkable net population growth when reaching their niche: the nitrate–ammonium transition zone. This common, yet understudied, geochemical transition zone represents an oasis in the sedimentary energetic desert, and the growth it supports is of major importance for the global nitrogen cycle.

The deep sedimentary biosphere is populated by microbes that once resided in the surface layers but over time became buried deeper and deeper into the sediments as new material settled on the seafloor (1). Below the bioturbation zone (generally <10 cm), these microbial cells are sealed off from a new supply of particulate material including organic carbon from the surface world (2). Despite the severe energy limitation, these cells can persist for millions of years and thousands of meters into the sediments (2, 3). Several lines of evidence show that the majority of the microbes are indeed alive (4) and active (5), albeit with extremely low metabolic rates (6, 7), conserving only enough energy to keep the cells in a state of maintenance, e.g., repairing DNA damage and maintaining vital cell functions (2). In this scenario there is no growth (net biomass production) but rather the cells are turning over their own biomass and slowly replacing themselves at estimated rates on the order of 10 to thousands of years (8, 9). Although microbial growth has been observed ex situ in laboratory incubations (10, 11) and sometimes assumed (1, 2), direct evidence of in situ net growth in the marine deep biosphere is missing.

Energy availability is one of the most fundamental factors limiting life; however, it has not been explicitly demonstrated to control microbial abundances in the deep biosphere (12). The global trend of decreasing total cell abundances with sediment depth (13) is typically explained by reduction in energy availability over time, leading to a net decay of biomass (14). While it stands to reason that the deep sedimentary realm generally can be viewed as an energetic desert with diminishing energy following increasing sediment depth/age, a number of geochemical transition zones (GTZs) with higher energy density exist. Typical GTZs include the sulfate–methane transition zones (15) and the oxic–anoxic transition zones (16), which both represent oases for microbial cells where energy from redox reactions can be harvested through specific metabolic pathways. These zones are also known to harbor a higher standing stock of microbes than adjacent depths and the presumed intensified redox reactions have been invoked to explain this phenomenon (15, 16). Whether this theory can be generalized to other GTZs, however, is still unknown, and a clear link between energy availability and growth in these zones is also missing.

Here we present the geochemistry, microbial ecology, and energetics of anammox bacteria in the understudied nitrate–ammonium transition zone (NATZ), which marks the transition of the dominant porewater N species from nitrate to ammonium. We provide strong indications for in situ growth of anammox bacteria and link the growth to increased power supply in ∼80,000-y-old subsurface sediments. Through genomic characterization of the dominant anammox bacterium, we also reveal the potential features that enable them to survive and grow in the subsurface. By consuming most of the upward flux of ammonium from deep anoxic sediments, the growth and activities of sedimentary anammox bacteria and their associated organisms have important implications for the global fluxes of nitrogen between marine sediments and the overlying ocean.

## Results and Discussion

### Widespread Occurrence of Sedimentary NATZ.

**Fig. 1.**
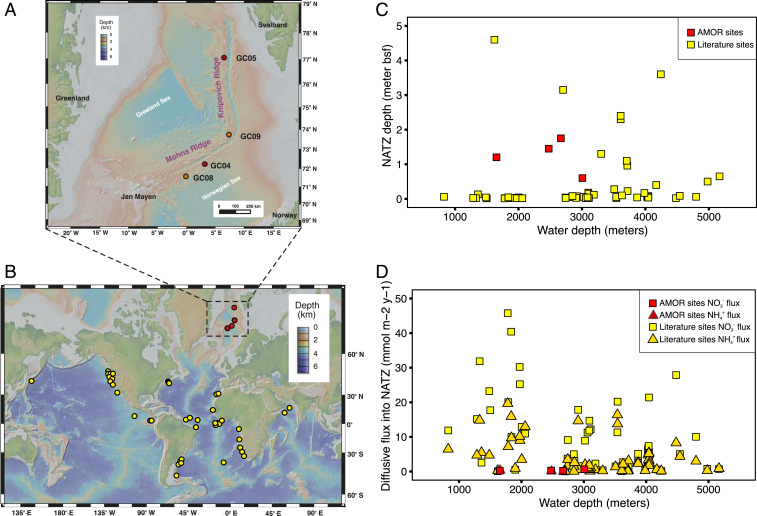
Study sites and global occurrence of NATZ in marine sediments. (*A*) Bathymetry map of the Arctic Mid-Ocean Ridge in the Norwegian-Greenland Sea. Cores GC08 and GC09 (orange) were sampled during summer 2014. Cores GC04 and GC05 (red) were sampled during summer 2016. (*B*) Location of marine sediments bearing an observed NATZ, identified based on previously published profiles of nitrate and ammonium. Maps in *A* and *B* were created in GeoMapApp version 3.6.10 using the default Global Multi-Resolution Topography Synthesis basemap. (*C*) Plot of NATZ depth against water depth of sediment locations included in *B*. (*D*) Plot of diffusive fluxes of nitrate and ammonium into NATZ against water depths for sediment locations included in *B*.

**Fig. 2.**
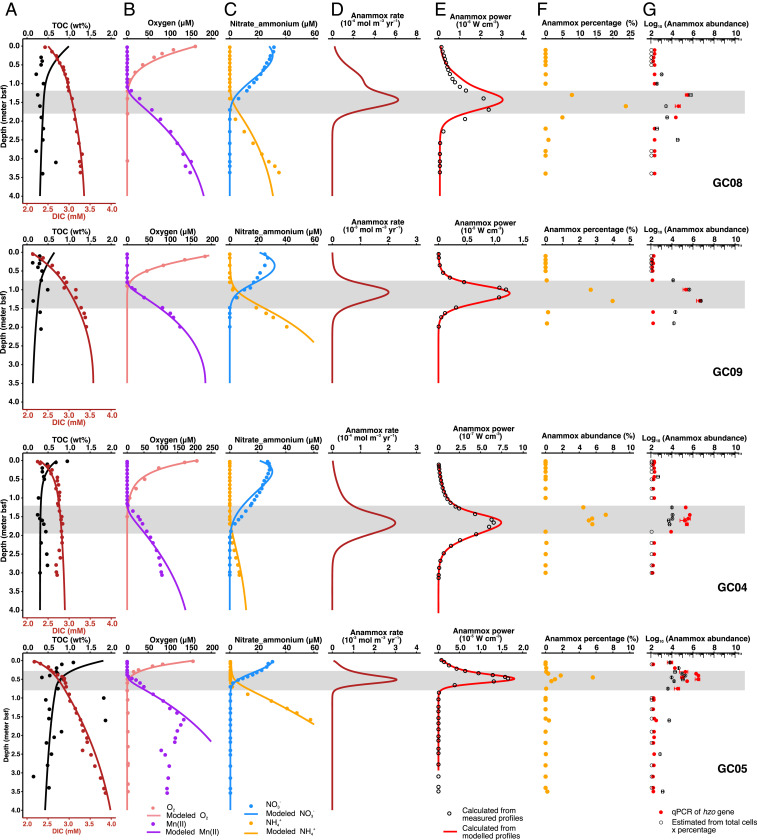
Environmental context, reaction rate, power supply, and distribution of anammox bacteria in AMOR sediment cores. (*A*–*C*) Measured (dots) and modeled (lines) depth profiles of TOC, DIC, oxygen, dissolved manganese, nitrate, and ammonium. (*D*) Anammox rate calculated based on model simulation. Note different *x*-axis scales used between cores. (*E*) Power supply of anammox calculated as the products of anammox rate and Gibbs free energy per anammox reaction (calculated from measured [dots] and modeled [lines] concentrations of relevant chemical species) presented in *SI Appendix*, Fig. S3. Note different *x*-axis scales used between cores. (*F*) Percentage of anammox bacteria from the genus of *Scalindua* in the amplicon libraries. (*G*) Anammox bacteria abundance quantified by two methods: 1) qPCR targeting the *hzo* gene (encodes the hydrazine dehydrogenase) (filled red dots), and 2) estimated as the product of anammox bacteria percentage in *F* and the total cell abundances quantified by 16S rRNA gene copies (open dots). Gene abundances below detection limit were arbitrarily shown as 100 copies g^−1^. Error bars represent the SD of triplicate qPCR measurements. The NATZ in each core is highlighted by a gray band.

We retrieved four sediment cores (2.0 to 3.6 m long) from the seabed of the Arctic Mid-Ocean Ridge (AMOR) at water depths of 1,653 to 3,007 m (Fig. 1*A* and *SI Appendix*, Table S1) and performed geochemical measurements and microbiological analyses with high vertical resolution. Although the cores are separated by more than 300 nautical miles, they exhibited similar geochemical profiles (Fig. 2 *A*–*C* and Dataset S1) summarized as follows: 1) O_2_ monotonically decreased and was depleted at a depth of 0.4 to 1.2 m below seafloor (mbsf), while dissolved Mn^2+^ built up right below the oxygen depletion depths; 2) NO_3_^−^ was abundant in the oxic zone and depleted in layers below the oxygen depletion depth; and 3) NH_4_^+^ was abundant in the deep anoxic sediment but undetectable in the oxic zone. These geochemical profiles all displayed a well-defined NATZ, where nitrate diffusing downward from the oxic zone and ammonium diffusing upward from deeper anoxic sediments are coconsumed, presumably by the anammox process (Fig. 2 and *SI Appendix*, Table S1). In order to investigate the global occurrence of this particular GTZ, we searched through geochemical profiles from earlier studies (see *SI Appendix* for details) and identified the NATZ at 63 additional sites (Fig. 1*B* and see also *SI Appendix*, Fig. S1 for the porewater nitrate and ammonium profiles). These sites were mainly located on continental slopes and midocean ridges of the Atlantic, Pacific, and Indian oceans (Fig. 1*B*) over a wide water depth range of 900 to 5,200 m. The NATZ depth in these sediments varies between 1.3 and 460 cm bsf, with no clear relationship to water depths (Fig. 1*C*). These data suggest that the NATZ is a widespread GTZ in the vast, yet discretely sampled, deep sea sedimentary realm.

### The NATZ Is the Primary Location for Anammox.

We applied a one-dimensional reaction-transport model (17) to simulate the geochemical profiles and calculate the rates of various reactions, including anammox (see *SI Appendix*, Table S2 for the reaction equations and rate expressions) in the four AMOR cores. The applied boundary conditions (*SI Appendix*, Table S3) and model parameters (*SI Appendix*, Table S4), allowed a simulation matching the measured profiles of total organic carbon (TOC), dissolved inorganic carbon (DIC), O_2_, NO_3_^−^, NH_4_^+^, and Mn^2+^ (Fig. 2 *A*–*C* and see also *SI Appendix*, Fig. S2 for detailed comparisons). In particular, as indicated by root mean square errors (*SI Appendix*, Table S5), the modeled concentrations of NO_3_^−^ and NH_4_^+^ showed only minor deviations from the measured ones (1.1 to 3.1 μM for NO_3_^−^ and 0.5 to 4.6 μM for NH_4_^+^), suggesting that the modeled profiles and reaction rates provided a realistic estimation of the in situ nitrogen cycling processes (see also *Model parameterization and sensitivity analysis* in *SI Appendix*, *Supplementary Text*). Our model predicts that anammox is restricted to the NATZs across all of the four cores (Fig. 2*D*), although from a thermodynamic perspective this process is exergonic at most depths (with Gibbs free energy of up to 200 kJ mol^−1^ N; *SI Appendix*, Fig. S3). This confined window of anammox in the NATZ is due to the combination of the coexisting reactants (NH_4_^+^ and NO_2_^−^/NO_3_^−^) and the absence of oxygen inhibition.

In our compiled dataset of sediment sites (*n* = 67) with an NATZ, 56 sites (84% of the total sites, including GC04 and GC08 from AMOR) have no upward ammonium efflux from the NATZs (Fig. 2*C* and *SI Appendix*, Fig. S1), suggesting that most of the ammonium from deep anoxic sediments is being consumed there. At these sites, the downward fluxes of NO_3_^−^ are higher than the corresponding upward diffusive flux of NH_4_^+^ (Fig. 1*D*), which could provide sufficient electron acceptors to anammox bacteria to consume all of the upward diffusing NH_4_^+^, if denitrifying bacteria in the NATZ are efficient to reduce nitrate to nitrite. We argue that the catabolic and anabolic activities of the microorganisms inhabiting the NATZ especially anammox bacteria play a critical role in preventing ammonium flux from marine sediments to the deep ocean.

### Elevated Abundances of Anammox Bacteria of Low Diversity in NATZs.

**Fig. 3.**
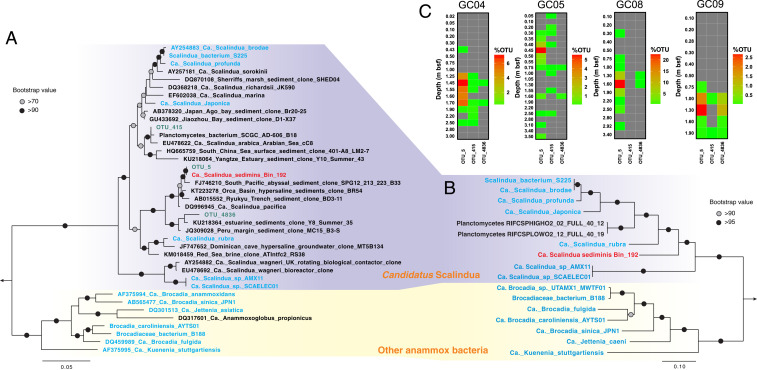
Phylogeny and vertical distribution pattern of *Scalindua* bacteria in AMOR sediments. (*A*) Maximum-likelihood phylogenetic tree of 16S rRNA genes of anammox bacteria. Sequences of the three *Scalindua* OTUs recovered from the sediments via 16S rRNA gene amplicon sequencing are shown in green. (*B*) Maximum-likelihood phylogenetic tree of anammox bacteria inferred from 14 concatenated ribosomal proteins. In both *A* and *B*, *Ca. S. sediminis* is highlighted in red, while other anammox bacteria are highlighted in green. *Paludisphaera borealis* PX4 and *Isosphaera pallida* were used as the outgroup for both trees. Bootstrap values higher than 70 and 90 are shown on nodes with open and filled circles, respectively. The scale bars correspond to estimated substitution per site. (*C*) Distribution of *Scalindua* OTUs in the four sediment cores. Depths of sediment horizons (meters below seafloor) are indicated on the vertical axis for each core. Note different scales are used for the anammox OTU percentages (of total community) between cores.

To study the microbial communities inhabiting these sediments, we performed 16S rRNA gene amplicon sequencing of all sampled sediment horizons (*n* = 66) from the four AMOR cores (*SI Appendix*, Fig. S4). We found that all sequences affiliated with known anammox bacteria belonged to the genus *Scalindua* and are represented by only three OTUs (operational taxonomic units, 97% identity), suggesting the presence of an anammox population with low diversity (Fig. 3 *A* and *C*). Consistent with the predicted anammox rate, *Scalindua* exhibited the highest relative abundance in the 16S rRNA gene libraries from the NATZ (accounting for up to 24% of the total community) in all cores, but was not detected in the upper oxic zone and only occasionally detected (<0.1%) below the NATZs (Fig. 2*F*). Similar peak occurrences of anammox bacteria in zones with counteropposing gradients of ammonium and nitrite/nitrate were also reported in anoxic water columns of the Mediterranean ocean (18), Black Sea (19), and the Golfo Dulce, Costa Rica (20). To exclude the possibility that these relative abundance peaks in the NATZs are the result of a closed compositional dataset (decrease/decay of other microbial taxa), we quantified the absolute abundances of anammox bacteria throughout the four cores by two methods: 1) quantitative PCR targeting the *hzo* gene encoding the hydrazine dehydrogenase, a marker that has been successfully used to characterize the vertical distribution of *Scalindua* in oxygen minimum zones (e.g., refs. 21, 22); and 2) calculating their abundance by multiplying the relative abundance of *Scalindua* in the total community by the total cell numbers determined by 16S rRNA gene quantification. Irrespective of the method used, the absolute abundances of anammox bacteria showed distinct maxima in the NATZ across all cores (Fig. 2*G*), consistent with the relative abundance profiles (Fig. 2*F*). A four order of magnitude increase of anammox bacteria abundance was consistently detected in the four cores by both methods (Fig. 2*G*), although deviations in exact abundances given by the two methods were noted especially in GC04 and GC05, likely due to the different copy numbers of 16S rRNA and *hzo* genes in anammox bacteria genomes.

### Increased Anammox Population Size Associated with Higher Power Availability.

To explore the factors driving the increases of anammox abundance in the subsurface NATZs, we calculated the power supply delivered through the anammox process as the product of the Gibbs energy (calculated using both measured and modeled nutrient concentrations) and the modeled rate of anammox (12). Similar to the distribution pattern of anammox reaction rates and anammox bacteria abundances, the power supply from both the measured and modeled data agree with each other and are highest in the NATZ across all cores (Fig. 2*E*), consistent with a higher standing stock of anammox bacteria in this geochemical transition zone.

**Fig. 4.**
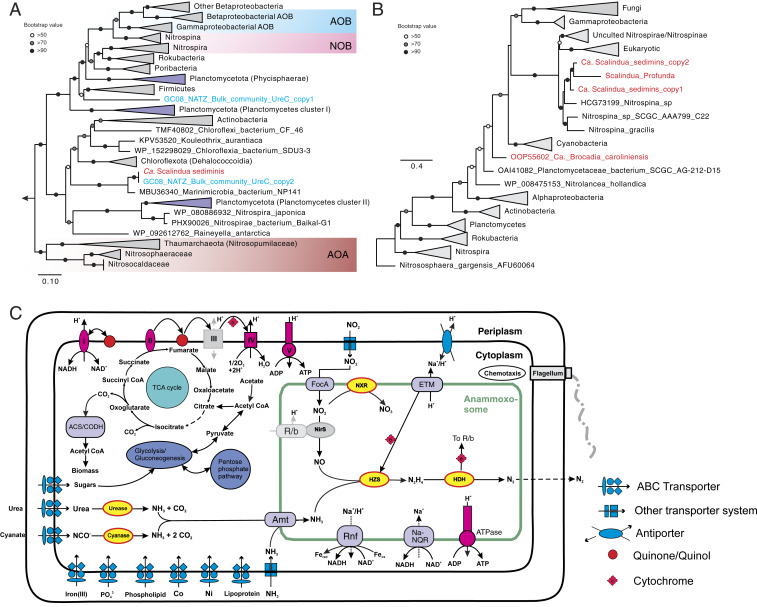
Metabolic potential of “*Ca. S. sediminis*”. (*A*) Maximum-likelihood phylogenetic tree of UreC (the catalytic alpha subunit of urease). The two UreC sequences detected in the metagenome assembly before binning are shown in blue and the one of *Ca. S. sediminis* is highlighted in red. Clades of nitrifying groups (i.e., AOA, AOB, and NOB) are highlighted in the shaded boxes, while sequences and clades of sequences of other Planctomycetes are shown in light purple. Bootstrap values of >50 are shown with symbols listed in the legend. The scale bar shows estimated sequence substitutions per residue. (*B*) Maximum-likelihood phylogenetic tree of cyanate hydratase (*cynS*) amino acid sequences (∼150 aa). The two copies of CynS detected in the *Ca. S. sediminis* genome and other anammox bacteria are highlighted in red. (*C*) Reconstruction of cell metabolic pathways based on the “*Ca. S. sediminis*” genome annotation. Enzyme complexes of the electron transport chain are labeled with Roman numerals. The flow of electron transfer is represented by blue arrows. HZS, hydrazine synthase; HDH, hydrazine dehydrogenase; HAO, hydroxylamine oxidase; NXR, nitrite oxidoreductase; TCA cycle, tricarboxylic acid cycle; Amt, ammonium transporter; ETM, electron transport module; FocA, nitrite/formate transporter; Rnf, ferredoxin-NAD:oxidoreductase; ACS/CODH, Acetyl-CoA synthase/carbon monoxide dehydrogenase, or Wood–Ljungdahl pathway; Na-NQR, Na-translocating NADH-quinone. Modules not detected in the genome annotation are shown in gray. Not drawn to scale. Uncertainties also exist for the position and size of the anammoxosome in the cells.

Given our current knowledge of the deep subsurface, this observation suggests in situ growth due to the local increase of power availability. However, another scenario that could, in principle, explain a potential increase of anammox cells in the NATZs is cell migration enabled by flagellar motility, because the dominant anammox bacterium in the NATZ has the full gene sets for flagellum synthesis (Fig. 4*C*). To investigate whether these anammox bacteria have sufficient metabolic energy to fuel flagellar rotation, we estimated cell-specific metabolic rates from predicted bulk anammox rates divided by anammox bacteria abundances. Cell-specific metabolic rates of anammox bacteria in the NATZs of the four AMOR cores fell in the range of 10^−3^ to 10^−1^ fmol NH_4_^+^ cell^−1^ d^−1^ (*SI Appendix*, Fig. S5), which is one to four orders of magnitude lower than those measured in laboratory bioreactors for other anammox bacteria (23, 24), supporting the general notion that per cell metabolic activities of subsurface microbes are much slower than their laboratory counterparts. (e.g., refs. 7, 9, 25). As bacterial flagella are driven mainly by proton motive force (26), we converted the cell-specific metabolic rates to cell-specific proton pumping rate (28 to 2,800 protons cell^−1^ s^−1^; see *SI Appendix*, *Materials and Methods* for the calculation details). These levels of proton pumping rates are lower than those required (10^4^ to 10^5^ protons per second) for the rotation of a single bacterial flagellum in *Escherichia* *coli* (27), suggesting that sedimentary anammox bacteria may not have enough energy for flagellar rotation. Therefore, we argue that the increased abundance of anammox bacteria in the NATZs is a result of in situ growth rather than cell migration. Combining with the recently reported resilience of anammox bacteria in arid soils (28), our results indicate that these organisms are not only able to survive prolonged periods of suboptimal conditions, but also proliferate once they encounter more energetically favorable ones in NATZ sediments up to ∼80,000-y-old sediment (i.e., 160 cm deep sediment in GC04 with a sedimentation rate of 2 cm/Ky) (29).

### Genome of the Most Dominant Anammox Bacterium in the NATZ.

In order to study the ecophysiology and potential adaptation mechanisms of anammox bacteria in the subsurface, we performed metagenome sequencing and analysis for four selected depths [10 cm (oxic zone), 100 cm (oxic–anoxic transition), 160 cm (NATZ), and 250 cm (manganese [Mn] reduction zone)] of GC08, because the most prominent anammox relative abundance increase was observed in this core (Fig. 2*F*). Through metagenome assembly and binning, we recovered a draft genome of *Scalindua* (95.5% completion) from the GC08 NATZ (*SI Appendix*, Fig. S6*A*). This genome was ∼3.0 Mbp, with 2,879 coding sequences across 71 scaffolds, thus over 1 Mbp smaller than other known *Scalindua* genomes (*SI Appendix*, Table S6). The assembled 16S rRNA gene sequence (1,573 bp) of this genome has a 100% match with that of the dominant *Scalindua* (OTU_5) found in our 16S rRNA amplicon analysis (Fig. 3*A*), suggesting this genome represents the most dominant *Scalindua* bacterium in these sediments. The genome shares less than 90% 16S rRNA gene sequence identity and 74 to 81% of genomic ANI (average nucleotide identity) with previously characterized *Scalindua* species from other marine habitats, including *Candidatus Scalindua rubra* (30) and *Candidatus Scalindua AMX11* (31) from seawater, *Candidatus Scalindua japonica* (32) and *Candidatus Scalindua profunda* (33) enriched from coastal sediments. Its 16S rRNA gene sequence forms a monophyletic clade with *Candidatus Scalindua pacifica*, a genotype detected in coastal Bohai Sea sediments (34), and other uncultured *Scalindua* from marine sediments (Fig. 3*A*). Phylogenetic analyses of concatenated ribosomal proteins (Fig. 3*B*) and the hydrazine synthase alpha, beta, and gamma subunits (*SI Appendix*, Fig. S7) showed that this genome, together with sequences from other marine sediments, represents a lineage within the genus of *Scalindua*. We propose a provisional taxon name for this uncultivated anammox bacterium, “*Candidatus Scalindua sediminis*,” based on its origin and prevalence (see below) in deep marine sediments.

Consistent with the ecotype-specific pattern evidenced from the phylogenetic analyses, a global search using the 16S rRNA gene as a query against sequences from natural environments available in the National Center for Biotechnology Information (NCBI) short reads archive (*Materials and Methods*) showed that *Ca. S. sediminis*-like bacteria (97% 16S rRNA nucleotide identity) were present in 156 samples (as of January 2020), all of which were from marine sediments. The spatial distribution pattern of this *Scalindua* bacterium resembles that of NATZ, i.e., both were mainly found in continental slope sediments or midocean ridges in the Atlantic and Pacific oceans (*SI Appendix*, Fig. S8). This resemblance might be explained by the observation that *Ca. Scalindua sediminis* mainly lives in the NATZ (Fig. 2 *F* and *G*).

To get more knowledge about the physiological state of *Ca. S. Sediminis*, we calculated the index of replication (iRep), an algorithm that uses the slope of genome coverage between the origin of replication and the terminus to estimate the ratio of active replicating cells in a population (35). The resulting iRep value was 1.32 for *Ca. S. sediminis* in the NATZ (*SI Appendix*, Fig. S6*B*), suggesting that 32% of this population was actively replicating at the sampling time, assuming each cell replicates two copies of its genome during growth (35). Consistent with the amplicon sequencing and qPCR assays (Fig. 2 *E* and *F*), *Ca. S. sediminis* was virtually undetectable at the other three sediment depths (i.e., genome coverages of <1×; *SI Appendix*, Fig. S6*A*) and thus unfortunately prevented iRep calculations outside the NATZ (≥5× coverage is required) (*SI Appendix*, Fig. S6*B*). Recently, a seawater-derived *Scalindua* bacterium grown in a laboratory bioreactor also exhibited similar iRep values (iRep of ∼1.2 to 1.4) (36). In principle, iRep can be used to estimate replicating cell ratios for any prokaryotic population in any given sample where high-quality genomes and the associated metagenome data are available. This make it an attractive tool for extracting information about the average in situ physiological status of populations without having to perform tedious laboratory experiments and circumvents the problems related to ex situ measurements. However, it is worth noting that iRep values cannot be converted to conventional growth rates (i.e., abundance changes over a certain duration of time), because no time dimension is included and death is not accounted for. iRep provides a snapshot in time of the ratio of replicating cells in a given population consisting of many cells with likely different physiological status (e.g., replicating, maintaining, or decaying), and can theoretically show all possible relationships (e.g., positive, negative, or unrelated) with conventional time-course-based growth rate measurements, depending on the ratios of cells in different physiological states and their rates. Nonetheless, the replication potential indicated by the iRep of *Ca*. *S. sediminis* is consistent with the observed four orders of magnitude increases of anammox bacteria abundance in the NATZs.

### Metabolic Potential of Dominant *Scalindua*.

The *Ca. S. sediminis* genome has the core genetic machinery for anammox metabolism, including hydrazine synthesis from NO and NH_4_^+^ catalyzed by hydrazine synthase (HZS), and hydrazine degradation to dinitrogen gas (N_2_) by hydrazine dehydratases (five variants of HZO) (Fig. 4*C*). Although *cd1* cytochrome nitrite reductase (NirS) was missing in the genome, we detected a *Scalindua nirS* gene in an unbinned 18,718-bp contig in the bulk metagenome assembly from the GC08 NATZ with a similar coverage as the rest of the contigs of *Ca. S. sediminis* (*SI Appendix*, Fig. S9), suggesting this contig may be part of the missing proportion of the *Ca. S. sediminis* genome. Therefore, *Ca. S. sediminis* can potentially use this protein to reduce nitrite to nitric oxide (NO). It can use the Wood–Ljungdahl pathway reversely to fix CO_2_ (Fig. 4*C*). For substrate acquisition, it can transport nitrite into the intracellular environment (i.e., anammoxosome) using the nitrite/formate transporter (FocA) and transport ammonium using ammonium transporters (amt) encoded with five copies in its genome (Fig. 4*C*).

Notably, *Ca. S. sediminis* has the potential to utilize urea and cyanate, suggesting a versatile metabolic lifestyle. For the urea metabolism, it encodes a urease operon (UreABC) and a urea-specific ABC transporter, as well as several urease accessory proteins (UreDEFG) that could facilitate the transport and intracellular degradation of urea to NH_4_^+^ (Fig. 4). The phylogeny of UreC (urease alpha subunit) showed that *Ca. S. sediminis* forms a branch well separated from known urea-utilizing nitrifiers (e.g., Thaumarchaeota, ammonia- and nitrite-oxidizing bacteria [AOB and NOB]) and other uncultured Planctomycetes (Fig. 4*A*), suggesting that *Ca. S. sediminis* have acquired the urea-utilizing capacity independently from the known urea-utilizing nitrifying organisms and relatives in the phylum of Planctomycetes. For the cyanate metabolism, *Ca. S. sediminis* has two copies of cyanate hydratase (encoded by the *cynS* gene), catalyzing the degradation of cyanate to NH_4_^+^ and CO_2_ (Fig. 4). CynS phylogeny showed that the two CynS sequences are similar to those of *Ca. S. profunda* (37) and three *Nitrospina* genomes (Fig. 4*B*) but not to the genomes of other Planctomycetes, suggesting that either cyanases in the two *Scalindua* genomes may have similar evolutionary history or they were horizontally transferred from the same taxon. Urea and cyanate are two dissolved organic nitrogen compounds ubiquitously present in seawater (38) and also detected in marine sediment porewater (39). The utilization of these two compounds has been suggested for *Scalindua* lineages found in the pelagic oxygen minimum zones based on chemical measurements (40, 41) and supported by single-cell genome sequencing (42). Here we expand this observation to marine sediments, by unambiguously identifying a urease operon and two cyanases in a single *Scalindua* genome. These two metabolic traits may not only enable *Ca. S. sediminis* to have access to alternative energy sources (i.e., urea and cyanate), but also provide it with an internal source of ammonium allowing it to persist under the severe competition disadvantage relative to ammonia-oxidizing Thaumarchaeota (43) in the upper oxic sediment layers.

Among the available *Scalindua* genomes, *Ca. S. sediminis* is unique in encoding two operons of HZS, although two HZSs were also noticed in the complete genome of *Kuenenia stuttgartiensis* outside the genus of *Scalindua* (44). The two copies of the HZS operon are phylogenetically close to each other (*SI Appendix*, Fig. S7). Due to the gene-dose effect (i.e., the copies of a particular gene present in a genome, known to be related to gene expression levels) (45), they may be more efficient to facilitate elevated turnover of ammonium derived from the degradation of urea and cyanate.

The anammox bacteria population dominated by *Ca. S. sediminis* has to persist for up to 80,000 y in sediments above the NATZ where the anammox metabolism is not favorable and therefore are likely to employ other metabolic strategies to stay alive. The *Ca. S. sediminis* genome has a cytochrome *cbb3* menaquinol oxidase, a feature that also exists in other anammox genomes (e.g., refs. 31, 32, 44), which could enable it to persist in partly oxygenated environments such as the upper section of sediment columns. In addition to the common F-type adenosine triphosphate (ATP) synthase, *Ca. S. sediminis* has an additional ATP synthase: V-type ATPase, only present in *Candidatus Brocadia sinica* among the other known anammox bacteria and may be configured into the electron transport chain linked to organic matter utilization. Compared to the other five existing *Scalindua* draft genomes, *Ca. S. sediminis* is enriched in genes involved in transport and metabolism of amino acids, nucleotides, coenzymes, and lipids (*SI Appendix*, Fig. S10). Because *Ca. S. sediminis* has the complete pathway for glycolysis, the near-complete the tricarboxylic acid (TCA) cycle (only fumarate hydratase missing) and the electron-transport chain for respiration (consisting of complexes I, II, III, IV (cytochrome *cbb3* menaquinol oxidase), and V-type ATPase) (Fig. 4*C* and Dataset S2), it is possible that this bacterium can degrade organic acid using nitrate as the electron acceptor, as observed in other characterized anammox bacteria (46–48). This capacity may provide an additional and probably crucial fitness advantage, allowing it to conserve energy when local conditions are not favorable for conventional anammox.

### Microbial Nitrogen Cycling in the NATZ.

**Fig. 5.**
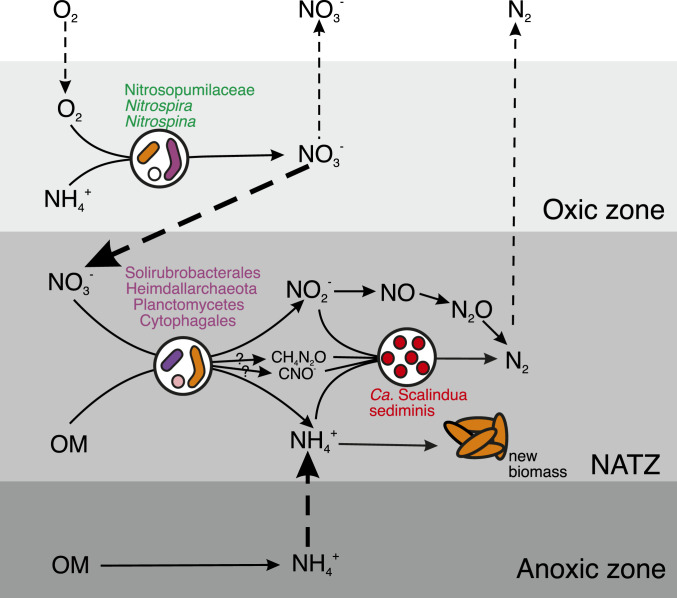
Conceptual model of *Ca. Scalindua* thriving in the NATZ and their dependencies on other microbial guilds and processes. Metabolism of *Ca. Scalindua* depends on both reduced nitrogen (ammonium) and oxidized nitrogen (nitrite). The ammonium source of *Ca. Scalindua* in the NATZ is mainly from the upward diffusive flux of NH_4_^+^ from the anoxic zone, while local organic matter degradation by denitrifying bacteria and urea and cyanate degradation of *Ca. Scalindua* can also release NH_4_^+^. For the nitrite source, *Ca. Scalindua* probably rely on the activity of nitrate-reducing bacteria, which generates nitrite by reducing nitrate, mainly produced by nitrifiers in the overlying oxic zone and diffusing downward into the NATZ. In addition to the anammox reaction, nitrite can be consumed by denitrifying bacteria and ammonium can be assimilated to generate new biomass by local organisms such as *Ca. S. sediminis*. OM, organic matter.

The canonical anammox metabolism requires both NH_4_^+^ and NO_2_^−^, which are probably mainly supplied by other organisms. By analyzing both 16S rRNA gene amplicon sequencing and the metagenomic data, we provided a conceptual model on the dependencies and interactions between nitrogen cycling microbes in the NATZ at our sites (Fig. 5). While abundant NH_4_^+^ are steadily available to *Scalindua* in the NATZ, porewater NO_2_^−^ was not detectable throughout the sediment cores, indicating that the *Scalindua* bacteria may depend on either ammonia oxidizers or nitrate reducers in close proximity within the NATZ to provide nitrite. Although low abundances of ammonia-oxidizing archaea were also observed in sediments below the oxic zone (*SI Appendix*, Fig. S4), they have no known genetic machinery to use alternative electron acceptors other than O_2_ (49), questioning their contributions to nitrite production in the anoxic NATZ. Instead, we found a variety of periplasmic nitrate reductase alpha subunit (NarG) sequences affiliated with Planctomycetes, Heimdallarchaeota, Cytophagales (Bacteroidetes phylum), and Solirubrobacterales (Actinobacteria phylum) in the metagenome assembly of the NATZ of GC08 (*SI Appendix*, Fig. S11*A*). Using qPCR, we also detected 10^4^ to 10^6^ copies g^−1^ of *narG* gene in the NATZs of all four AMOR cores (*SI Appendix*, Fig. S11 *B*–*D*), comparable to anammox abundances. These *narG*-bearing nitrate-reducing bacteria could reduce nitrate to nitrite by using organic matter as electron donor and fulfill most of the nitrite demand of *Scalindua* (Fig. 5). The nitrate consumed in the NATZ was derived from the overlying oxic zone where nitrifiers including ammonia-oxidizing Nitrospumilaceae and nitrite-oxidizing *Nitrospira* and *Nitrospina* are abundant (*SI Appendix*, Fig. S4). The presence of *nirS* and *nirK* genes encoding nitrite reductases (*SI Appendix*, Fig. S11 *B*–*D*) suggests that a proportion of nitrite could be reduced further to gaseous nitrogen (Fig. 5). Further enrichments and cocultures are required to confirm these proposed microbial interactions in the sedimentary NATZ.

## Conclusions

We show that the sedimentary NATZ, a widespread yet overlooked GTZ, is a hotspot for ongoing anammox. By consuming most of the upward flux of NH_4_^+^ from deep anoxic sediments, anammox bacteria and the associated organisms, especially denitrifiers in NATZs, play an important role in controlling the ammonium flux out of the seafloor. We provide compelling evidence for in situ net growth of anammox bacteria in this zone after being exposed to highly unfavorable conditions during the 80,000-y transit from the seafloor to the NATZ. Growth was quantitatively linked to increased availability of power of anammox, demonstrating that the generally growth-arrested subseafloor microbes can be reactivated to proliferate in situ in this energy-limited biosphere. Future explorations with sediment cores of higher vertical resolution (e.g., capturing the exact depths where anammox bacteria start to grow) and integrating the microbial traits into existing reaction-transport models (e.g., ref. 50) will lead to more quantitative insights into the cellular power threshold above which anammox bacteria can replicate. The predominant *Ca. S. sediminis* has genomic features that enable it to use alternative energy sources (e.g., urea and cyanate) and adapt to energy-limiting conditions. Experimental characterization is needed to confirm the importance of these alternative substrates and their interactions with the heterotrophic denitrifiers in the same zone. Considering the widespread occurrence of NATZs (Fig. 1*B*) and other GTZs (16, 51), net growth is expected to occur ubiquitously for various microbial guilds within their ideal niches in the marine deep biosphere.

## Materials and Methods

### Study Area and Sampling.

Sediment cores used in this study were retrieved using a gravity corer from the seabed of the AMOR with water depths of 1,653 to 3,007 m, during the CGB Summer Cruise 2014 (GC08 and GC09) and 2016 (GC04 and GC05) onboard the Norwegian R/V *G.O. Sars*. GC04 (3.1 m long; 2,668-m water depth) and GC05 (3.5 m long; 3,007-m water depth) were collected from the middle section of the Knipovich Ridge, while GC08 (3.4 m long; 2,476-m water depth) and GC09 (2.0 m long; 1,653-m water depth) were collected from the central and northeastern ends of the Mohns Ridge, respectively (Fig. 1*A* and *SI Appendix*, Table S1). Oxygen concentrations were measured immediately using a needle-type fiber-optic oxygen microsensor (optodes, PreSens) inserted manually into sediments. Pore water extractions were conducted with Rhizons samplers at 5-cm intervals over the first half meter and at 25- or 30-cm intervals below that depth. Subsamples for microbiological analysis were taken from the same depths as porewater extraction by using sterile 10-mL cutoff syringes. Details about onboard core handling, porewater analysis, and onshore solid phase analyses are provided in *SI Appendix*, *Materials and Methods*.

### Reaction-Transport Modeling.

We used a one-dimensional reaction transport model (17) to simulate the depth profiles of relevant porewater solutes and solid phase organic carbon content. The explicitly modeled chemical species include oxygen, nitrate, ammonium, Mn(II), DIC in aqueous phase, TOC (expressed in weight percent [wt%]), and manganese oxide (MnO_2_) in the solid phase. The model considers two sets of reactions: 1) primary reactions during organic matter degradation: aerobic degradation (*R*_1_), heterotrophic denitrification (*R*_2_), and MnO_2_ reduction (*R*_3_); as well as 2) secondary reactions, including nitrification (*R*_4_), Mn(II) oxidation with oxygen (*R*_5_), and anammox (*R*_6_). Model simulations assume chemical species, including all implicit reactive intermediates, are at steady state. The applied upper and lower boundary conditions are listed in *SI Appendix*, Table S3. Model parameters (*SI Appendix*, Table S4) were calibrated by visually comparing the model simulation outputs against measured depth profiles of O_2_, NH_4_^+^, NO_3_^−^, DIC, Mn(II), and TOC, the combination of which was suggested to be sufficient to constrain this type of model with reasonable precision (52). Detailed information on model structure, reaction rate expressions, and relevant parameters can be found in *SI Appendix*, *Materials and Methods*.

### Global Occurrence of NATZ in Global Marine Sediments.

Geochemical profiles of porewater nitrate and ammonium indicating a NATZ in marine sediments were either retrieved from published literature or obtained from the PANGAEA database (https://www.pangaea.de/). Porewater profiles were manually checked and discarded if they 1) do not reach the nitrate-depletion depth (i.e., approximately the NATZ) or 2) contain too few datapoints (<6).

### Calculation of Gibbs Free Energy and Power Supply of Anammox.

The standard Gibbs free energy (*∆G*_*r*_^*0*^) was calculated using thermodynamic data of standard Gibbs free energy of formation of each reactant/product, corrected to near in situ pressure and temperature using the R package *CHNOSZ* (53). The Gibbs free energy of anammox (*∆G*_*r*_) was then calculated following the description in LaRowe and Amend (12). To provide a contentious prediction, modeled concentrations of NH_4_^+^ and NO_3_^−^ were used, while measured concentrations were also used for discrete depths to assess the potential uncertainty. Following the notion proposed in LaRowe and Amend (12), the power supply of anammox was calculated as the product of the Gibbs free energy and reaction rate of anammox (predicted by the reaction-transport model).

### DNA Extraction and Gene Quantifications.

DNA for amplicon sequencing and qPCR was extracted from ∼0.5 g of sediment per sample using the PowerLyze DNA extraction kits (MOBIO Laboratories, Inc.) with minor modifications. Amplicon libraries of the 16S rRNA gene were prepared using a two-round PCR amplification strategy with the primers of 515F/806r, as described in Zhao et al. (16). Details about amplicon preparation and sequence analysis are provided in *SI Appendix*, *Materials and Methods*. For quantification of anammox bacteria, PCR amplification was performed for the *hzsA* gene using primer set hzsA_1597A/hzsA_1857R and the thermal cycling condition described in ref. 54 as well as the *hzo* gene using hzoF1/hzoR1 (55). PCR products were only obtained from the latter amplification. Therefore, the abundance of anammox bacteria was quantified using qPCR by targeting the *hzo* gene, although this assay may overestimate anammox cell abundances due to the multiple copies of *hzo* in anammox genomes (e.g., refs. 32, 54 and five variants in *Ca. S. sediminis*). The abundance of denitrifying bacteria was quantified by targeting the *narG* (encoding the periplasmic NarG), *nirS* and *nirK* genes (encoding the cytochrome cd1-and Cu-containing nitrite reductases, respectively), using the protocol described in Zhao et al. (16). In addition, the abundances of archaeal and bacterial 16S rRNA genes were also quantified and used to estimate total and anammox cell abundances. Detailed information can be found in *SI Appendix*, *Materials and Methods*.

### Metagenomic Sequencing, Binning, and Annotation.

Metagenomic libraries were constructed using a Nextera DNA Flex Library Prep kit (Illumina) and sequenced (2 × 150 bp) by an Illumina HiSeq 2500 sequencer. The quality of the reads and presence of adaptor sequences were first checked using FastQC v.0.11.5 (56) and then processed with Trimmomatic v.0.36 (57). Quality-controlled paired-end reads were de novo assembled into contigs using Megahit v.1.1.2 (58) with the k-mer length varying from 27 to 117. Contigs larger than 1,000 bp were automatically binned with MaxBin2 v2.2.5 (59) using default parameters. The genome bin of *Ca. S. sediminis* was manually refined using the gbtools (60) based on the GC content, taxonomic assignments, and differential coverages in different samples. The quality of the resulting *Scalindua* genome was checked using the CheckM v.1.0.7 “lineage_wf” command, based on the Planctomycetes marker gene set (automatically selected by CheckM). Genes in the genome of *Ca. S. sediminis* were predicted using Prodigal (61). Genome annotation was conducted using Prokka v.1.13 (62), eggNOG (63), and BlastKoala (64) using the Kyoto Encyclopedia of Genes and Genomes (KEGG) database. Functional assignments of genes of interest were confirmed using BLASTp against the NCBI RefSeq database. Metabolic pathways were reconstructed using KEGG Mapper (65). Detailed information on the metagenome data analysis can be found in *SI Appendix*, *Materials and Methods*.

### Phylogenetic Analyses.

All available high-quality anammox bacterial genomes were downloaded from the NCBI database and were included in the phylogenomic analysis, which was based on marker genes consisting of 14 syntenic ribosomal proteins (rpL2, 3, 4, 5, 6, 14, 16, 18, 22 and rpS3, 8, 10, 17, 19), demonstrated to undergo limited lateral gene transfer (66). These selected proteins, among the conservative single-copy ribosomal proteins included in Campbell et al. (67), were identified in Anvi’o v.5.4 (68) using hidden Markov model (HMM) profiles. Sequences were aligned individually using MUSCLE (69), and alignment gaps were removed using trimAl (70) with the mode of “automated.” Individual alignments of ribosomal proteins were concatenated. The maximal likelihood phylogenetic tree was reconstructed using IQ-TREE v.1.5.5 (71) with the best-fit model selected by ModelFinder (72), and 1,000 ultrafast boostrap iterations using UFBoot2 (73).

A maximum-likelihood phylogenetic tree based on 16S rRNA genes was also constructed for known anammox bacteria and close relatives of the three *Scalindua* OTUs identified via BLASTn (74) in the NCBI database. Sequences were aligned using MAFFT-LINSi (75) and the maximum-likelihood phylogenetic tree was inferred using IQ-TREE with the procedure described above. Detailed information on the phylogenetic analysis of functional proteins, including the three subunits of hydrazine synthase (HzsA, HzsB, and HzsC), urease alpha subunit (UreC), cyanate dehydrogenase (CynS), respiratory NarG, and cd1 cytochrome nitrite reductase (NirS) are provided in *SI Appendix*, *Materials and Methods*.

### Comparative Genomic Analysis of *Scalindua.*

Genomes of *Ca. S. rubra* (30), *Candidatus Scalindua brodae* (76), *Ca. S. japonica* (32), *Ca. S. profunda* (33), *Ca. S. AMX11* (31), and *Ca. S. sediminis* (recovered in this study) were included in the comparative genomic analysis using Anvi’o v.5.4 (68) according to the workflow described at merenlab.org/2016/11/08/pangenomics-v2/. All genomes were annotated using Prokka v.1.13 (62) and BLASTp using the clusters of orthologous groups of proteins (COG) (77) as the reference database. Specific metabolic characteristics inferred from the annotations of genes with known homologs and identified with the pangenomic analysis are discussed in the main text.

### iRep Calculation.

The iRep is a measure of the average genome copy number across populations of cells (35). This method is based on the observation that during growth the replication of prokaryote circular genomes generally occur bidirectionally from a fixed origin to a fixed terminus (opposite the origin) (78). In iRep, the positions of the origin and the terminus are estimated by sorting genome fragments (metagenome-assembled genome scaffolds) from highest to lowest coverage based on coverage across overlapping fragments (or windows). Theoretically, the slope of the resulting read recruitment sine curve should be reflective of the ratio of replicating cells in the whole population, with steeper slopes indicating higher ratios of replication cells.

In this study, the genome quality of *Ca. S. sediminis* passed the quality criteria of iRep calculation (i.e., >75% completed, >5 coverage, and ≤175 scaffolds per Mbp of sequence) (28), iRep was successfully calculated in the NATZ of GC08 (160 cm bsf). Such a calculation was not performed in the other three depths because genome coverages were too low. Scaffolds <5 kb were discarded prior to reads mapping, which did not change the genome quality given by CheckM. Reads mapping files generated using Bowtie2 (79) were used to calculate iRep using the default settings without GC corrections (28).

### Global Distribution of *Ca. S. sediminis-*like Anammox.

The occurrence of *Ca. S. sediminis*-like anammox in natural environments was assessed using IMNGS (80) against the available short read archive (SRA) datasets in the NCBI database with the full-length 16S rRNA gene sequence as query. Reads were counted as matching reads if they 1) are longer than 200 bp and 2) show >97% nucleotide sequence identity to the query. Samples with fewer than 10 matching reads were discarded. Only natural environments with more than 0.01% matching reads were included.

## Supplementary Material

Supplementary File

Supplementary File

Supplementary File

## Data Availability

All sequencing data used in this study are available in the NCBI Short Reads Archive under the project no. PRJNA529480. In particular, raw metagenomic sequencing data are available in the NCBI database under the following BioSamples (SAMN11268098, SAMN11268104, SAMN11268106, and SAMN11268109). The *Ca. S. sediminis* genome is available under the accession no. SAMN12415826. Raw geochemical data can be found in Dataset S2. The R script of the reaction-transport model is available at GitHub (https://github.com/ruizhao087/Reaction-Transport-Model-for-marine-sediments). All other study data are included in the article and supporting information.
