# Supplementary material for: Geochemical transition zone powering microbial growth in subsurface sediments
Source: Proc Natl Acad Sci U S A. 2020 Dec 7;117(51):32617–26. doi: 10.1073/pnas.2005917117 (PMC7768721; doi:10.1073/pnas.2005917117)
Supplement: Supplementary File [file pnas.2005917117.sapp.pdf]

PNAS
www.pnas.org

Supplementary Information for

Geochemical transition zone powering microbial growth in subsurface sediments

Rui Zhao[1,2*], José M. Mogollón[3], Sophie S. Abby[4,+], Christa Schleper[4], Jennifer F. Biddle[2], Desiree Roerdink[1], Ingunn H. Thorseth[1], and Steffen L. Jørgensen[1*]

* Correspondence: Rui Zhao, ruizhao@udel.edu Or Steffen L. Jørgensen, steffen.jorgensen@uib.no

This PDF file includes:

Supplementary Text
Figures S1 to S12
Tables S1 to S6
Legends for Datasets S1-S2
SI References

Other supplementary materials for this manuscript include the following:

Datasets S1
Datasets S2

www.pnas.org/cgi/doi/10.1073/pnas.2005917117

Supplementary Text

Model parameterization and sensitivity analysis

Due to the environmental and spatial heterogeneities of marine sediments, model parameters can vary over many orders of magnitude [*e.g.* (1, 2)]. These parameters include kinetic-related rate constants that drive internal transformations and boundary conditions (*e.g.* fluxes) that control inputs and outputs from the system. While most parameters used in our model were similar to those applied in reaction-transport modeling of other deep-sea sediments (3-6), the degradation constants of the most labile organic matter (kfox), were significantly lower than those used in the above mentioned studies (Table S4). This is not surprising, as the reactivity of organic carbon is very complex and depends on a range of factors that include the chemical nature of organic compounds together with the biogeochemical characteristics of the hosting environment (7). For this reason, a thorough review of modeling studies for organic matter degradation kinetics found very limited transferability of its kinetic parameters across sites (1). We performed a sensitivity analysis, by varying kfox in the range of 10-fold to 1/10-fold of the baseline value (6.9e-5 yr^{-1}), to verify our choice of this particular parameter. As shown in Fig. S12, increasing or decreasing the kfox value can dramatically change the simulation results and failed to reproduce the measured porewater profiles, while using the baseline value (used in the model results present in Fig. 2) can produce good matches with the measured porewater profiles.

From this sensitivity analysis, it is also obvious that varying a single parameter can cause significant changes of multiple model simulation results, due to the nature of the intertwined reactions considered in the model. While it is possible to use Monte Carlo analysis (8) or similar quantitative goodness to estimate the uncertainties for models fitting individual profiles, it is not practical to do so for models that are calibrated based on multiple measured profiles, because it is difficult to determine (1) which profile(s) should be prioritized in the error minimization and (2) the distribution range of each parameter over which to perform the random sampling. Therefore, our model parameters were largely determined by visual comparisons of the best possible fits of all the fix measured solutes/solids profiles (TOC, DIC, O_2, Mn(II), NO_3^-, and NH_4^+), as previous studies using similar model architectures (3-5, 9). This follows as each profile inherently contains both a measurement and a modeling error (based on the numerical representation of its processes and its subsequent parameterization), which could be normalized

with weighting procedures, but that would inherently introduce a bias. Instead, we calculated the root mean square error (RMSE) of the modeled and measured profiles of O_2, Mn(II), NO_3^-, NH_4^+, and DIC in the four AMOR cores. RMSEs of NO_3^- and NH_4^+ support that our model provides a good simulation of the N cycling processes in these sediments (Table S5).

The modeled TOC concentrations failed to match with those measured values at the most surface sediments, which could be due to the absences or measurement errors of TOC in samples in the uppermost 10-30 cm (Fig. 2a). In addition, the exponentially-decreasing TOC profiles given by our model also deviated from the discrete elevations of measured TOC in the subsurface (Fig. 2a). These TOC elevations may represent temporal changes of organic matter fluxes over millennia and are difficult to capture by our model, in which organic matter deposition was assumed to operate with a constant flux (*i.e.* at steady state).

Potential measurement errors and error propagation

Due to limited amounts of extracted porewater and multiple solutes to be measured, only one measurement was done for each of the geochemical items (See the supplementary data S2 for the comprehensive raw data). Also, the O_2 measurement is extremely vulnerable to potential air intrusion during the measurement. Therefore, the O_2 concentration measurement at each depth was performed only one time in a short duration to prioritize accurate profiles. However, because the measured geochemical profiles were mainly used to calibrate the reaction-transport model and to predict the NATZ position and first-order estimates of anammox reaction rates, the unconstrained measurement errors could not substantially affect the model predictions. If measurement errors only occur for depths where solutes are measureable, our measured profiles can provide reliable indications where solutes are not measurable. For instance, nitrate-depletion depth can be easily recognized, which is always associated with NATZ and anammox rate maxima, as indicated by the sensitivity analysis.

All gene abundances were determined in triplicate via qPCR, and the standard deviations are presented using horizontal error bars in Fig. 2g and Fig. S11. The standard deviation of the qPCR propagates to cell-specific metabolic rates; however, all within the same order of magnitude and should not affect the overall conclusions on cell-specific rates.

Materials and Methods

Study area, sampling, and geochemical measurements

Sediment cores used in this study were retrieved using a gravity corer from the seabed of the Arctic Mid-Ocean Ridge (AMOR) with water depths of 1653 – 3007 m, during the CGB Summer Cruise 2014 (GC08 and GC09) and 2016 (GC04 and GC05) onboard the Norwegian *R/V G.O. Sars*. GC04 (3.1-m long; 2,668 m water depth) and GC05 (3.5-m long; 3,007 m water depth) were collected from the middle section of the Knipovich Ridge, while GC08 (3.4-m long; 2,476 m water depth) and GC09 (2.0-m long; 1,653 m water depth) were collected from the central and northeastern end of the Mohns Ridge, respectively (Figure 1a and Table S1). Cores were taken from areas without known hydrothermal activity. Retrieved cores were immediately sectioned into 1.5-m-long whole round cores and split in halves upon arriving on deck. Oxygen concentrations were measured immediately using a needle-type fiber-optic oxygen microsensor (PreSens, Regensburg, Germany) inserted manually into the sediments. The optode sensors were connected to a MICROX TX3 single channel fibre-optic oxygen meter, which was calibrated according to the manufacturer's protocols (PreSens, Regensberg, Germany). Pore water extractions were conducted with Rhizons samplers (10), from each of the 5 cm interval in the first half meter and 25 or 30 cm interval below that depth. Microbiology subsamples were taken simultaneously with porewater extraction, by using sterile 10 ml cut-off syringes from nearly identical depths as the porewater extraction, and immediately frozen at -80°C for onshore-based DNA analysis.

Nutrient concentrations in porewater were measured onboard. Concentrations of ammonium (NH_4^+), nitrate (NO_3^-) and dissolved inorganic carbon (DIC) were analyzed colorimetrically by a Quaatro continuous flow analyzer (SEAL Analytical Ltd, Southampton, UK), following the manufacturer's protocol. The photometric indophenol method was used for ammonium measurement (11). Nitrate was reduced to nitrite by a Cu-Cd reduction coil, and detected as a red complex (12). The protocol for DIC was based on ref. (13). Chloride (Cl^-) and sulfate (SO_4^{-2}) were measured by a Metrohm ion chromatography system. Porewater samples for metal concentrations (including Mn(II) and Fe(II)) were acidified by ultrapure nitric acid to a final concentration of 3 vol%, and stored in acid-cleaned HDPE bottles at 4°C until analysis. Metal concentrations were determined by Thermo Scientific iCap 7600 ICP-AES (inductively coupled plasma atomic emission spectrometry) at the University of Bergen. Quantification was done by

external calibration curves (multi element standard solutions prepared from certified single element solutions from Spectrapure) and Sc was used for internal standardization. For quality control and monitoring the performance during the analytical runs, the synthetic water CRM SPS-SW-2 (Spectrapure Standards AS) was analyzed repeatedly through the run. For additional control an in-house seawater standard was used.

Porosity was calculated as the weight loss of 1 cm^{-3} sediment after drying at 95^oC for 24 hours, assuming a dry sediment density of 1.6 g cm^{-3}. Dried sediments were also used for total organic carbon (TOC) and nitrogen (TON) measurements on an element analyzer (Analytikjena multi EA® 4000, Jena, Germany), after inorganic carbon removal by adding 1 mL of phosphoric acid.

Diffusive flux calculation

Diffusive fluxes of nitrate and ammonium into the NATZ of all cores were calculated based on the measured profiles using Fick's first law of diffusion:

$$J = \varphi \times D_s \times \delta[C]/\delta z$$

where, J is the flux; φ is the measured sediment porosity; D_s is sedimentary diffusion coefficient for a given solute ($m^2.yr^{-1}$) calculated using the *R* package *marelac* (14); z is the sediment depth below the seafloor (m); and $\delta[C]/\delta z$ equals the solute (NO_3^- or NH_4^+) concentration gradient ($mmol.m^{-3}$), calculated from nearby three data points.

Reaction-transport modeling

We used a one-dimensional reaction transport model (3, 4) to simulate the depth profiles of relevant solutes in porewater and organic carbon content in solid phase. In this study, the species explicitly modeled include oxygen, nitrate, ammonium, Mn(II), and dissolved inorganic carbon (DIC) in aqueous phase, and total organic carbon (TOC, expressed in weight percent wt%) and manganese oxide (MnO_2) in the solid phase. The model considers two sets of reactions: 1) the primary reactions involved in the organic matter degradation: aerobic degradation (R_1), heterotrophic denitrification (R_2), and MnO_2 reduction (R_3); 2) and the secondary reactions including nitrification (R_4), Mn(II) oxidation with oxygen (R_5) and anammox (R_6).

Model simulations assume that the geochemical profiles, including all implicit reactive intermediates, are near steady state.

Organic matter in the model was regarded to consist of 3 discrete components (the so-called 3-G model(15)), with the first two as the reactive ones and the third one as non-reactive. Aerobic respiration (R_1) was considered as the most favorable pathway of organic matter consumption, followed by heterotrophic denitrification (R_2) that is limited by nitrate concentration and inhibited by oxygen, and MnO_2 reduction (R_3) that is limited by MnO_2 concentration and inhibited by both oxygen and nitrate, implemented through serial inhibition terms (3). Secondary reactions (R_4-R_6) were represented through bimolecular kinetics, except for anammox which was also inhibited by oxygen. As nitrite is a highly active intermediate of multiple N cycle pathways, it was not explicitly simulated due to its rapid reactivity. Therefore, the model assumes the anammox reaction to be a reaction between NH_4^+ and NO_3^- following Mogollon et al (3). The C/N stoichiometry of the degraded organic matter was taken as the TOC/TON ratio. Diffusion coefficients were calculated as a function of the temperature (1 ^oC) and salinity (35 Practical Salinity Unit (PSU)) using the R package *marelac* (14). As boundary conditions (Table 3), the model is constrained by fixed concentrations of O_2, NH_4^+, NO_3^-, DIC, Mn(II), and fixed organic matter flux at the sediment-water interface, and zero gradient conditions at the lower boundary of the sediment domain indicated in Table S3. The remaining model parameters (Table S4) were calibrated by comparing the model simulation outputs against the measured depth profiles of O_2, NH_4^+, NO_3^-, DIC, Mn(II), and TOC (Fig. 2) until satisfied visual fits for all profiles were reached.

The numerical solution for the partial differential equations was implemented in R following the approach outlined in Soetaert and Meysman (16). In short, the spatial derivatives of the partial differential equations were expanded as a finite difference grid (200 equidistant layers over the sediment domain of 10 cm). After discretization, the resulting set of ordinary differential equations was integrated using the stiff equation solver *ode* implemented in R through the *deSolve* package (17). We calculate the root mean square error (RMSE), the square root of the sum of the squared differences between modeled and measured values, for O_2, Mn(II), NO_3^-, NH_4^+, and DIC, to assess the goodness of our model simulations.

Global occurrence of NATZ in global marine sediments

Geochemical profiles indicating a nitrate-ammonium transition zone (NATZ) in marine sediments (*i.e.,* the narrow overlap interval where downward diffusing nitrate encounter the upward diffusing ammonium) were previously reported in the literature [*e.g.,* (18-31)]. Nitrate and ammonium profiles were obtained directly from these publications using the online tool WebPlotDigitizer (http://automeris.io/WebPlotDigitizer), when not available in public databases. Additional sediment nitrate and ammonium profiles were obtained from the PANGAEA database (www.pangaea.de) by searching using a combination of the following key words: "marine sediment", "ammonium", and "nitrate". Porewater profiles of ammonium and nitrate were manually checked and those containing too few datapoints (<6) were discarded. All sites harboring a clear NATZ were included in the global map prepared using GeoMapApp (32).

Calculation of Gibbs free energy and power supply of anammox

The standard Gibbs free energy (ΔG_r^0) was calculated using the thermodynamic data of standard Gibbs free energy of formation of each reactant/product that corrected to near *in situ* pressure and temperature in the R package *CHNOSZ* (33). Gibbs free energy of anammox (ΔG_r) was then calculated following the description in LaRowe and Amend (34), using the equation:

$$\Delta G_r = \Delta G_r^0 + RT \times In Q_r$$

where Q_r refer to reaction quotient of anammox reaction, R represents the gas constant (*i.e.* 8.314 J mol^{-1} K^{-1}), and T denotes temperature in Kelvin. Both the measured profiles and the model fits were used in this calculation. Note that NH_4^+ concentrations above the NATZs and NO_3^- concentrations below the NATZs were below detection limit but were arbitrarily set at 0.01 μM to allow the calculation. N_2 concentrations in the sediment porewater were not measured, but assumed to be constant at 0.625 mM throughout the cores, according to a handful of N_2 concentration measurements in marine anoxic sediments (35, 36). Final values were expressed in kJ per mole of electron transferred, kJ $(mol\ e^-)^{-1}$, assuming six electrons transferred per anammox reaction.

Following the notion proposed in LaRowe and Amend (34), the power supply of anammox reaction, P_s, is calculated using the following equation:

$$P_s = \Delta G_r \times R_{anammox}$$

where ΔG_r is the Gibbs free energy of anammox, $R_{anammox}$ is the anammox rate predicted from the reaction-transport model.

DNA extraction

DNA for amplicon sequencing and qPCR was extracted from ~0.5 gram of sediment per sample using the PowerLyze DNA extraction kits (MOBIO Laboratories, Inc.) with the following minor modifications: 1) Lysis tubes were replaced by G2 tubes (Amplikon, Odense, Denmark), and 2) water bathed for 30 min at 60 ^{o}C prior to bead beating (speed 6.0 for 45 seconds) using a FastPrep-24 instrument (MP Biomedicals). A blank extraction was carried out in parallel with each extraction batch (including ~15-25 samples from the same core) following the same procedure without sediment addition. The DNA was eluted into 80 µL of molecular grade double-distilled H_2O (ddH_2O) and stored at -20 ^{o}C until analysis. DNA for metagenomic sequencing was extracted from ~ 7 g sediment (0.7 g sediment in 10 individual lysis tubes) of each of the four selected horizons in core GC08 following the procedure described above, except the final elution step: The DNA extracts from each sample were iteratively eluted from the 10 spin columns into 100 µL of ddH_2O for further analysis.

Amplicon sequencing and sequence analysis

16S rRNA genes were amplified using the primer pair 515F/806R in a two-round amplicon preparation, with an optimal PCR cycle number in the first round to minimize over-amplification., Amplicon libraries were sequenced on an Ion Torrent Personal Genome Machine. Sequencing reads were quality filtered and trimmed to 220 bp using the USEARCH pipeline (37) and chimera were detected and removed using UCHIME. Trimmed reads were clustered into operational taxonomy units (OTUs) at >97% nucleotide sequence identity using UPARSE (38). Most of the OTUs detected in the extraction blanks (negative controls) were manually removed, except for a few OTUs that may be introduced into the blanks by cross-contamination. Overall, >99.9% of reads in the negative controls were removed. Samples were subsampled to 20,000 reads for each sediment horizon. The taxonomic classification of OTUs was performed using the lowest common ancestor algorithm implemented in the CREST package (39) with the SilvaMod128 database (September 2016 release) as reference. The relative abundance of anammox bacteria was taken as

the sum of the percentages of *Candidatus* Scalindua OTUs in the total communities, and visualized in heatmaps generated using the *R* package *ggplot2* (40).

Quantification of total microbial community and anammox bacteria

Abundances of anammox bacteria was quantified using qPCR by targeting the *hzo* gene (encoding the hydrazine dehydrogenase responsible for the degradation of hydrazine to N_2) using the primer pair hzoF1/hzoR1 (41) following the procedure described elsewhere (6). The abundances of denitrifying bacteria were quantified by targeting the *narG* (coding the periplasmic nitrate reductase alpha subunit), *nirS* and *nirK* genes (coding cytochrome cd1- and Cu-containing nitrite reductases, respectively), using the protocol described in (6). In addition, archaeal and bacterial 16S rRNA genes were quantified as described in (42). Total cell abundance was estimated from 16S rRNA gene copies, assuming 5.0±2.9 copies of 16S rRNA genes for each bacterial genome, and 1.7±0.9 copies in each archaeal genome (43). Anammox abundance was also calculated as the product of the total cell abundance and the percentage of the genus of *Candidatus* Scalindua in the total community assessed by amplicon sequencing (see description below). All gene abundances were determined in triplicate by qPCR, and standard deviations are presented using horizontal error bars in Fig. 2g and Fig. S11.

Cell-specific rate of anammox

We estimated cell-specific metabolic rates from the bulk anammox rate given by the reaction-transport model divided by the anammox abundance measured by *hzo*-based qPCR. This calculation was limited to sediment horizons within the NATZ in each core, because anammox bacteria abundances were rarely detected outside the NATZ (Fig. 2g). To facilitate the direct comparison with *E. coli* (44), the cell-specific metabolic rate (in units of fmol NH_4^+ cell^{-1} d^{-1}) were converted to cell-specific proton pumping rate (in the unit of protons cell^{-1} s^{-1}), assuming four mols of protons were transferred per mol of NH_4^+ oxidized by anammox bacteria for establishing the proton motive force (45).

Metagenomic sequencing and analysis

DNA was sheared into 400 bp fragments using Covaris, and paired-end libraries were constructed using a Nextera DNA Flex Library Prep kit (Illumina). Metagenomic libraries were sequenced (2×150 bp) by an Illumina Hiseq 2500 sequencer at the Vienna Biocenter Core Facilities GmbH (Vienna, Austria). The quality of the reads and presence of adaptor sequences were checked using FastQC v.0.11.5 (46). Then the sequencing data were processed with Trimmomatic v.0.36 (47) to trim read-through adapters (ILLUMINACLIP:TruSeq2-PE.fasta:2:30:10), trim low quality base calls at the starts and ends of reads (LEADING:3, TRAILLING:3), remove reads that had average phred score lower than 25 in a sliding window of 10 bp (SLIDINWINDOW:10:25), and finally remove reads shorter than 100 bp (MINLEN:100). The overall quality of processed reads was evaluated in a final check with FastQC v.0.11.5, to ensure only high-quality reads were used in the downstream analysis.

Assembly and genome binning

The quality-controlled paired-end reads were *de novo* assembled into contigs using Megahit v.1.1.2 (48) with the k-mer length varying from 27 to 117. Contigs larger than 1000 bp were binned with MaxBin2 v2.2.5 (49) using the default parameters. The quality of the obtained genome bins was assessed using the option "lineage_wf" of CheckM v.1.0.7 (50), which uses lineage-specific sets of single-copy genes (SCGs) to estimate completeness and contamination and assigns contamination to strain heterogeneity if amino acid identity is >90%. Genome bins of >50% completeness were manually refined using the gbtools (51) based on the GC content, taxonomic assignments, and differential coverages in different samples. Coverages of contigs in each sample were determined by mapping trimmed reads onto the contigs using BBMap v.37.61 (52). Taxonomy of contigs were assigned according to the taxonomy of the single-copy marker genes in contigs identified using a script modified from blobology (53) and classified by BLASTn (54). SSU rRNA sequences in contigs were identified using Barrnap (55), and classified using VSEARCH (56) with the SILVA 132 release (57) as the reference. To improve the quality of the genome of *Ca.* Scalindua sedimins, the metagenome reads of the sample GC08_160cm were mapped onto the contigs using BBmap (52), and the aligned reads were re-assembled using SPAdes v.3.12.0 (58). After manual removal of contigs shorter than 1000 bp, the resulting scaffolds were visualized and re-binned using gbtools (59) as described above.

The quality of the resulting *Scalindua* genome was checked using the CheckM "lineage_wf" command again, based on the Planctomycetes marker gene set (automatically selected by CheckM).

Genome annotation

Genes in the genome of *Ca.* Scalindua sediminis were predicted using Prodigal (60). Genome annotation was conducted using Prokka v.1.13 (61), eggNOG (62), and BlastKoala (63) using the KEGG database. The functional assignments of genes of interest were also confirmed using BLASTp against the NCBI RefSeq database. Metabolic pathways were reconstructed using KEGG Mapper (64).

Phylogenetic analyses

All available high-quality anammox bacterial genomes were downloaded from the NCBI database and included in the phylogenomic analysis. The phylogenomic analysis was based on marker genes consisting of 14 syntenic ribosomal proteins (rpL2, 3, 4, 5, 6, 14, 15, 18, 22 and rpS3, 8, 10, 17, 19) that have been demonstrated to undergo limited lateral gene transfer (65). These selected proteins, among the conservative single-copy ribosomal proteins included in Campbell, *et al.* (66), were identified in Anvi'o v.5.4 (67) using Hidden Markov Model (HMM) profiles, following the procedure outlined at http://merenlab.org/2017/06/07/phylogenomics/. Sequences were aligned individually using MUSCLE (68), and alignment gaps were removed using trimAl (69) with the mode of "automated". Individual alignments of ribosomal proteins were concatenated. The maximal likelihood phylogenetic tree was reconstructed using IQ-TREE v.1.5.5 (70) with LG+F+I+G4 the best-fit model selected by ModelFinder (71), and 1000 ultrafast boostrap iterations using UFBoot2 (72) to assess the robustness of tree topology.

A maximum likelihood phylogenetic tree based on 16S rRNA genes was also constructed for known anammox bacteria and close relatives of the three *Scalindua* OTUs identified via BLASTn (54) in the NCBI database. Sequences were aligned using MAFFT-LINSi (73) and the maximum-likelihood phylogenetic tree was inferred using IQ-TREE v.1.5.5 with GTR+F+R3 as the best-fit substitution model and 1000 ultrafast bootstraps following the procedure described above.

For the phylogeny of HzsA (hydrazine synthase subunit alpha), the genomes of known anammox bacteria were downloaded from the NCBI database, annotated using Prokka v1.13 (61), and the HzsA

amino acid sequences were extracted. Additional HzsA sequences of uncultured anammox deposited in the NCBI database were also identified using BLASTp (54) using the HzsA sequence of *Ca.* S. sediminis as the query. Sequences were aligned using MAFFT-LINSi (73) and the maximum likelihood phylogenetic tree was inferred using IQ-TREE v.1.5.5 following the procedure described above.

For the phylogeny of UreC (urease alpha subunit, also the catalytic subunit), the sequence of *Ca.* S. sediminis was used as the query in the BLASTp (54) search in the NCBI database (>50% similarity were retained), to identify its close relatives. These sequences were aligned using MAFF-LINSi (73) with reference sequences from Koch, *et al.* (74), and complemented with known nitrifiers (*e.g.* ammonia-oxidizing bacteria (AOB) from the genera of *Nitrosospira*, *Nitrosomonas*, *Nitrososcoccus*, nitrite-oxidizing bacteria (NOB) from *Nitrospira* and *Nitrospina*, and ammonia-oxidizing archaea (AOA) from the Thaumarchaeota phylum). For the CynS encoding cyanase (*i.e.* cyanate dehydrogenase), the two CynS sequences of *Ca.* S. sediminis were aligned using MAFFT-LINSi (73) with reference sequences from Palatinszky, *et al.* (75) and their close relatives in GenBank identified via BLASTp given a similarity threshold of 50%. Both alignments were then trimmed using trimAl (69) with the mode of "automated". Maximum likelihood phylogenetic trees were reconstructed using IQ-TREE v.1.5.5 (70) with the LG+C20+F+G substitution model and 1,000 ultrafast bootstraps.

Since the cytochrome cd1-containing nitrite reductase (NirS) was missing in the *Ca.* Scalindua sediminis draft genome, we searched this protein by BLASTp using the NirS of *Ca.* Scalindua rubra as the query against the coding sequences of the bulk metagenome assembly of the NATZ of GC08. The only NirS hit from the bulk assembly was aligned using MAFFT-LINSi (73) with sequences from other anammox bacteria, and their close relatives identified by BLASTp. The alignment was trimmed using trimAl (69) with the mode of "automated" and then was used to infer the maximum-likelihood phylogenetic tree using IQ-TREE v.1.5.5 with WAG+I+G4 as the substitution model and 1,000 ultrafast bootstraps. All phylogenetic trees were visualized and branches were collapsed using FigTree (http://tree.bio.ed.ac.uk/publications/), prior to figure preparations in CorelDraw 2019.

Potential denitrifying bacteria in the NATZ of GC08

Denitrifying bacteria can provide nitrite to anammox bacteria by reducing nitrate to nitrite using the nitrate reductase. To explore the presence and diversity of potential denitrifying bacteria in the NATZ, we extracted the NarG (nitrate reductase alpha subunit) sequences from the Prokka annotation of the GC08 NATZ metagenome assembly, and used them as the queries in BLASTp to identify their close relatives in the NCBI database. All these sequences were combined with the NarG sequences of several MAGs recovered from the four metagenomes of GC08 (details about these MAGs will be presented in a separate paper), and the NarG/NxrA sequences of various denitrifiers and nitrite-oxidizing bacteria published in (76, 77). All sequences were aligned using MAFFT-LINSi (73), and alignment gaps were removed using trimAl (69) with the mode of "automated". The maximum-likelihood phylogenetic tree was reconstructed using IQ-TREE v.1.5.5 (70) with LG+R7 as the best-fit substitution model and 1,000 ultrafast bootstraps.

Comparative genomic analysis of *Scalindua*

Genomes of *Ca.* S. rubra (78), *Ca.* S. brodae (79), *Ca.* S. japonica (80), *Ca.* S. profunda (77), *Ca.* S. AMX11 (81), and *Ca.* S. sediminis (recovered in this study) were included in the comparative genomic analysis using Anvio v.5.4 (67) according to the workflow described at http://merenlab.org/2016/11/08/pangenomics-v2/. All genomes were annotated using Prokka v.1.13 (61) and BLASTp using the Clusters of Orthologous Groups of proteins (COG) (82) as the reference database. The specific metabolic characteristics inferred from the annotations of genes with known homologs, and identified with the pangenomic analysis are discussed in the main text.

Global distribution of *Ca.* S. sediminis-like anammox

The occurrence of *Ca.* Scalindua sediminis-like anammox in natural environments was assessed using IMNGS (83) with the full-length 16S rRNA gene sequence as query. Reads with length longer than 200 bp and nucleotide sequence identity higher than 97% to the query were included as matching reads. Samples with <10 matching reads were discarded. Only natural environments with a proportion of matching reads higher than 0.01% were included.

Supplementary Figures and Tables

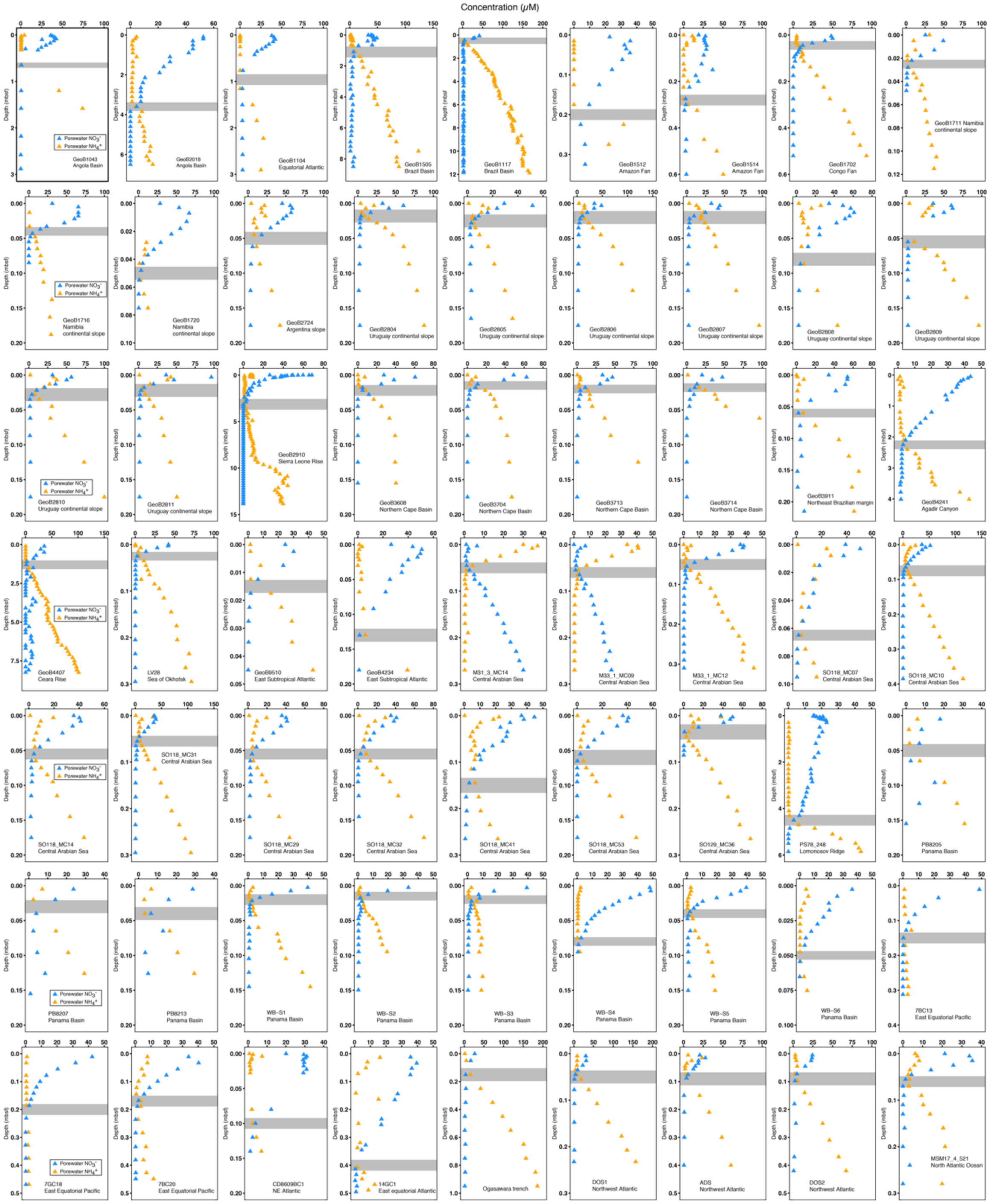

Fig. S1. Geochemical profiles of porewater nitrate (blue) and ammonium (orange) in sediment sites bearing a NATZ highlighted by grey bars.

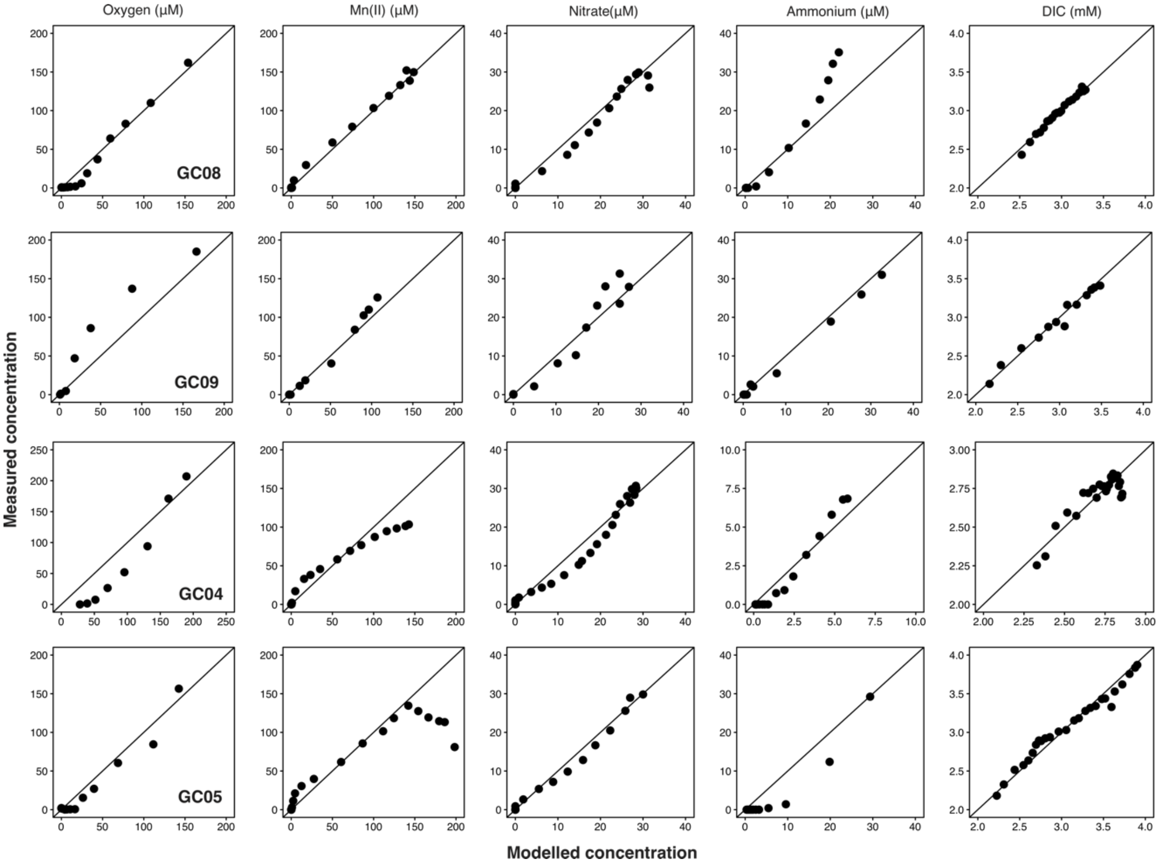

Fig. S2. Plots of modelled vs. measured porewater concentrations of oxygen, dissolved manganese, nitrate, ammonium, and dissolved inorganic carbon (DIC). In each plot, a reference line with a slope of 1 is also included.

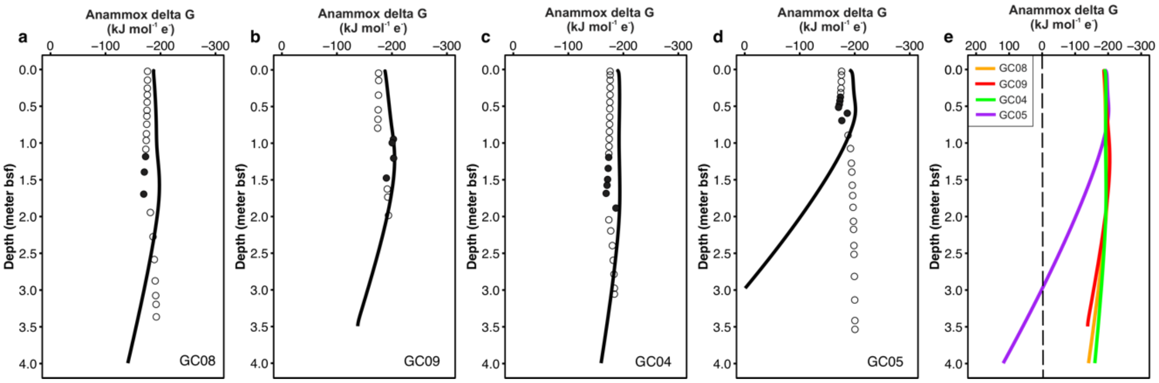

Fig. S3. Gibbs energy of the anammox reaction in the unit of kJ per mole of electron transfer. (a-d) Gibbs energy of the anammox reaction in individual cores. In each core, the solid line represents the calculation based on the model fits, while the circles denote those based on the measured profiles with the depths within the NATZ shown in filled circles. **(e)** A compilation of the model fits-based calculation of anammox Gibbs energy in all the four cores.

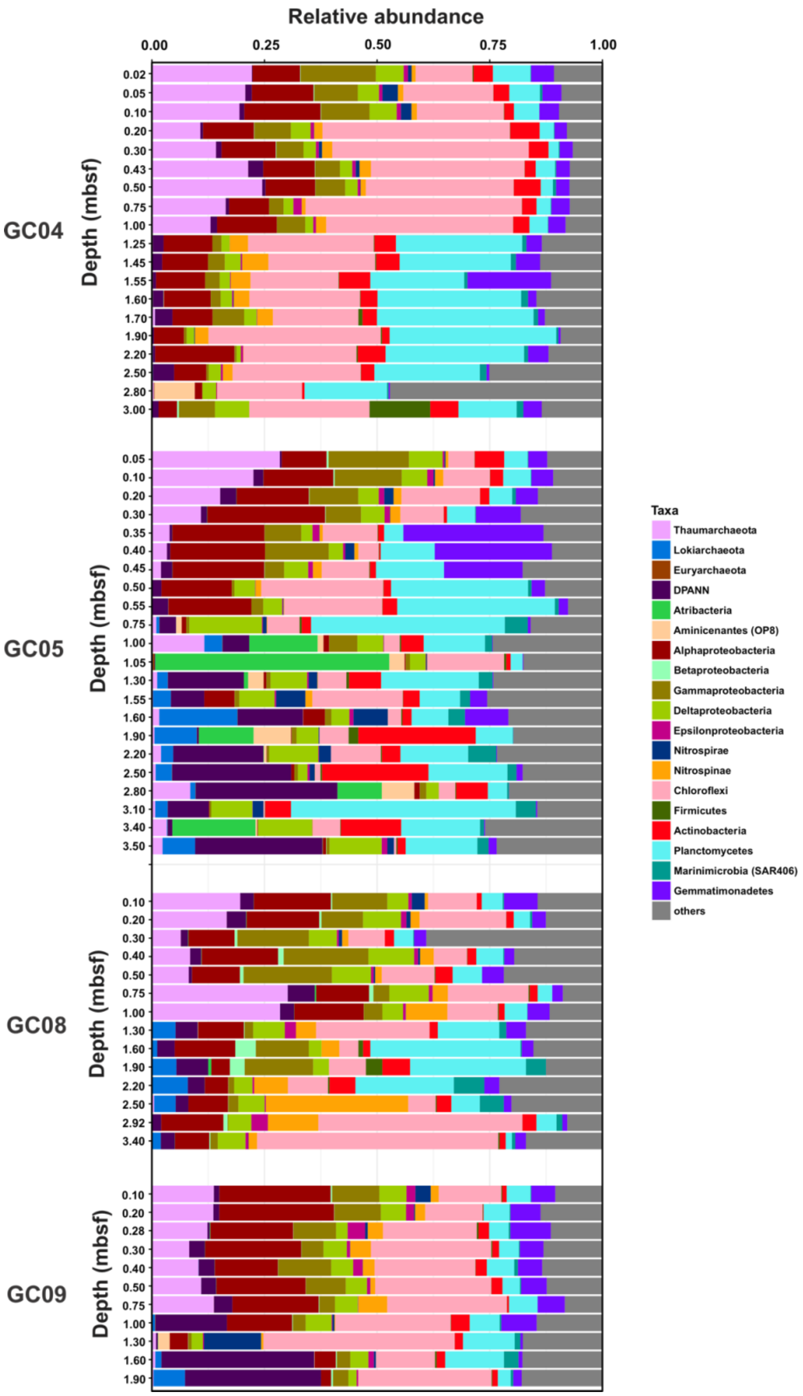

Fig. S4. Microbial communities inhabiting AMOR sediment cores assessed by 16S rRNA gene amplicon sequencing. Minor taxa were combined into the category of "others".

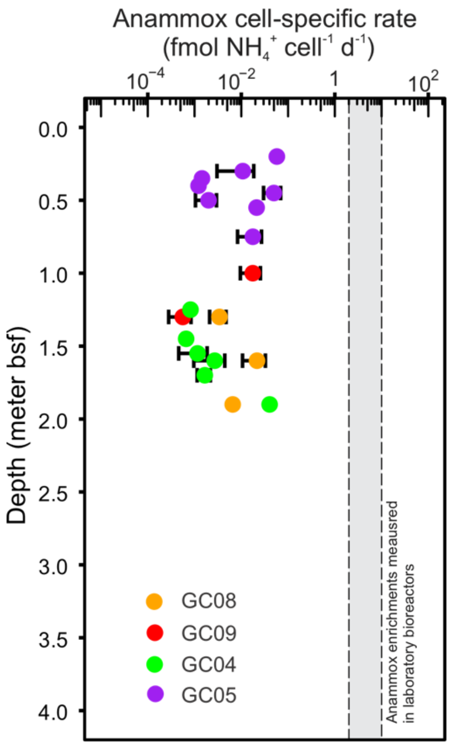

Fig. S5. Cell-specific rates of anammox bacteria in NATZ. Cell-specific rates of anammox were calculated by dividing the modeled bulk anammox reaction rate by the anammox cell number quantified by qPCR targeting the *hzo* gene. Error bars derived from triplicate quantification of anammox cell numbers using qPCR. The grey dashed box represents the cell-specific rate range (2-20 fmol cell^{-1} d^{-1}; from refs. (84, 85)) of anammox bacteria enrichments measured in laboratory reactors.

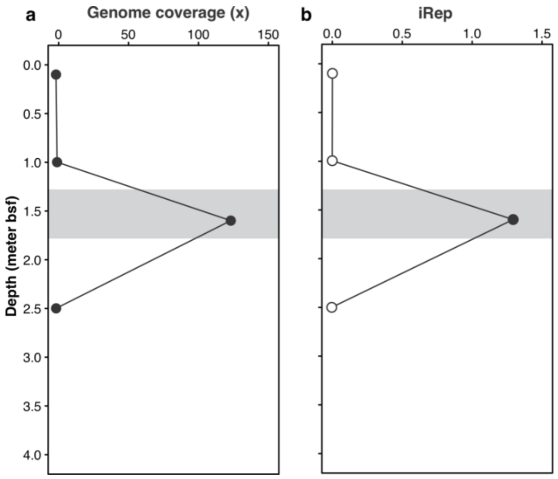

Fig. S6. Genome coverage (a) and index of replication (iRep, b) of *Ca.* Scalindua sediminis in GC08. In **(b)**, iRep calculation was only possible for the NATZ depth (160 cm) due to the low genome coverages in the other three depths (iRep in the other three depths were arbitrarily assigned to zero and marked with open circles). The NATZ was marked with a grey band in both panels.

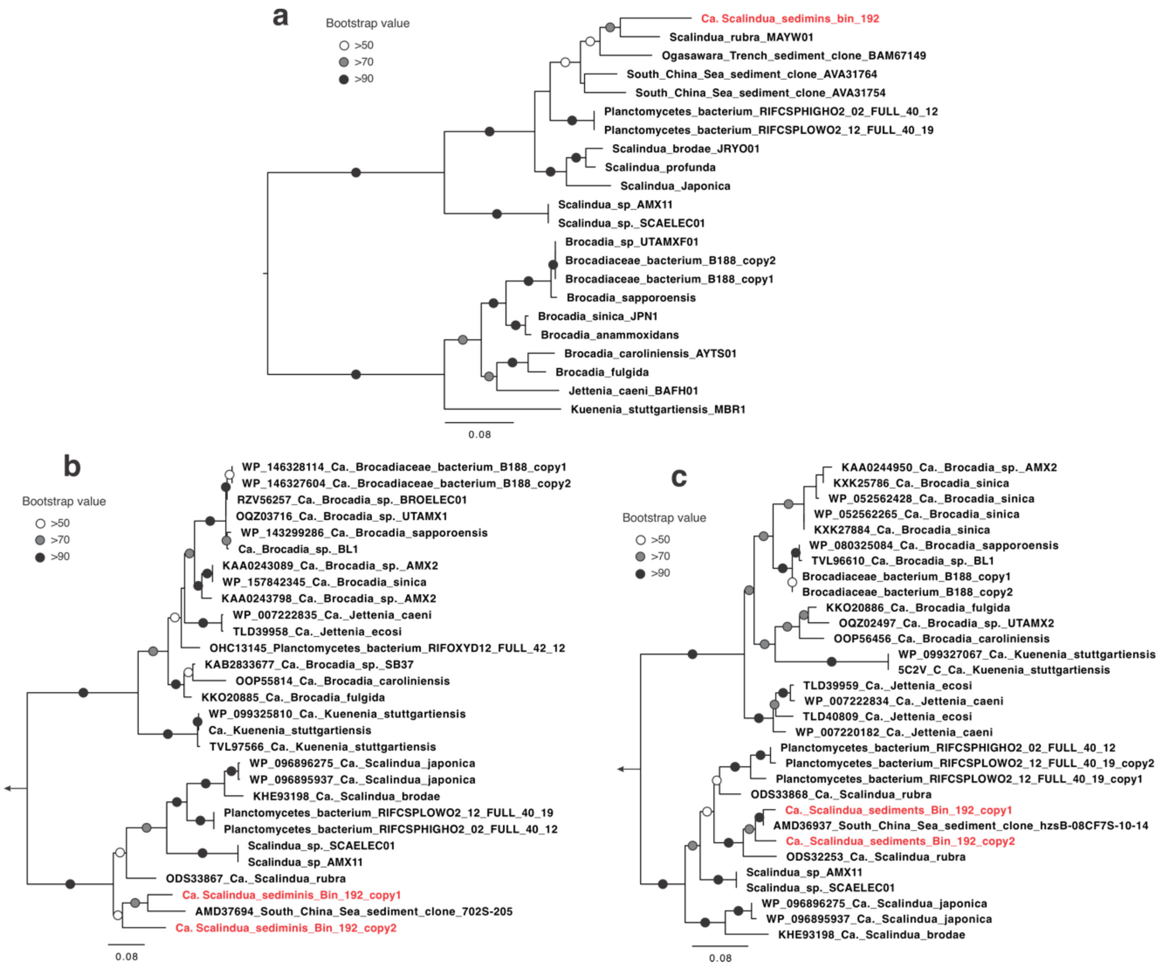

Fig. S7. Maximum-likelihood phylogenies of anammox hydrazine synthase alpha (a), beta (b), and gamma (c) subunits. All phylogenetic trees were reconstructed using IQ-TREE with 1000 fast bootstraps. Sequences of *Candidatus* Scalindua sediminis genome recovered in this study is highlighted in red. Bootstrap values of >50 are shown with symbols listed in the legend. The scale bars show estimated sequence substitutions per residue.

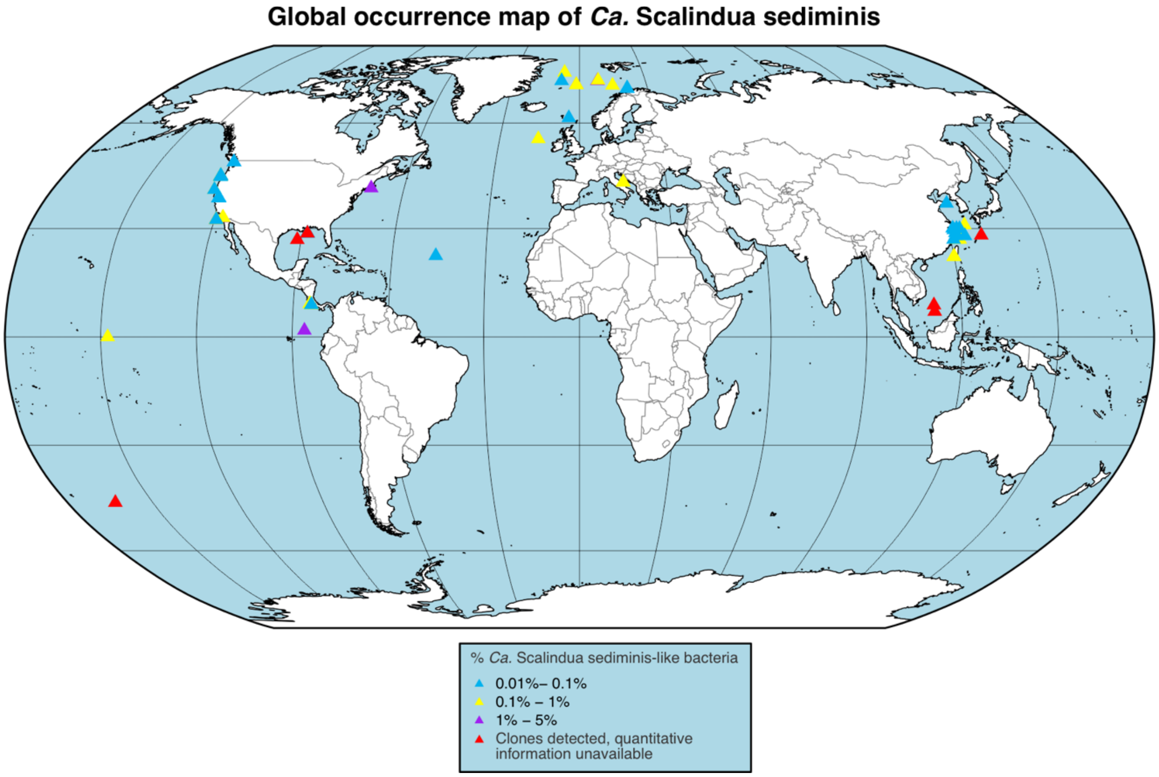

Fig. S8. Global distribution of the *Ca.* Scalindua sediminis-like bacteria. The map indicates sampling locations of public SRA datasets containing bacteria showed >97% similarity to the 16S rRNA gene sequence of *Ca.* Scalindua sediminis. Note that in some cases a single sampling site can represent multiple sediment depths. Relative abundance of *Ca.* Scalindua sediminis-like bacteria are shown in color-coded triangles.

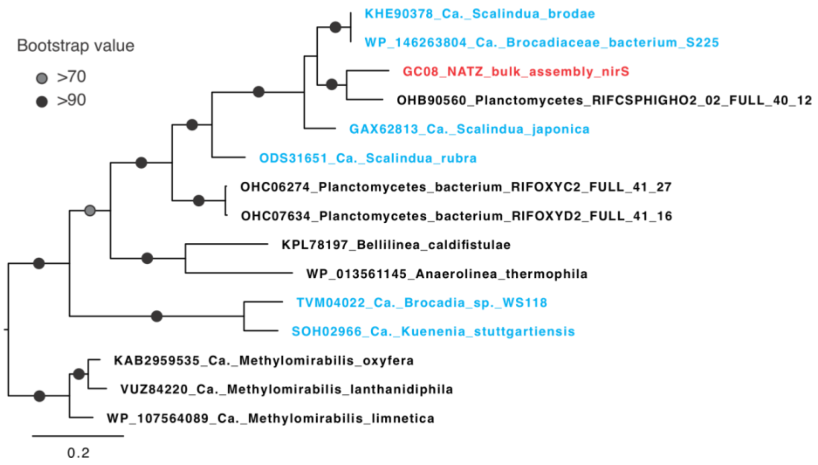

Fig. S9. Maximum likelihood phylogenetic analysis of cytochrome *cd1*-containing nitrite reductase (NirS) of anammox bacteria. The sequence from the metagenome assembly in the NATZ of GC08 is highlighted in red, while sequences of known anammox bacteria are shown in blue.

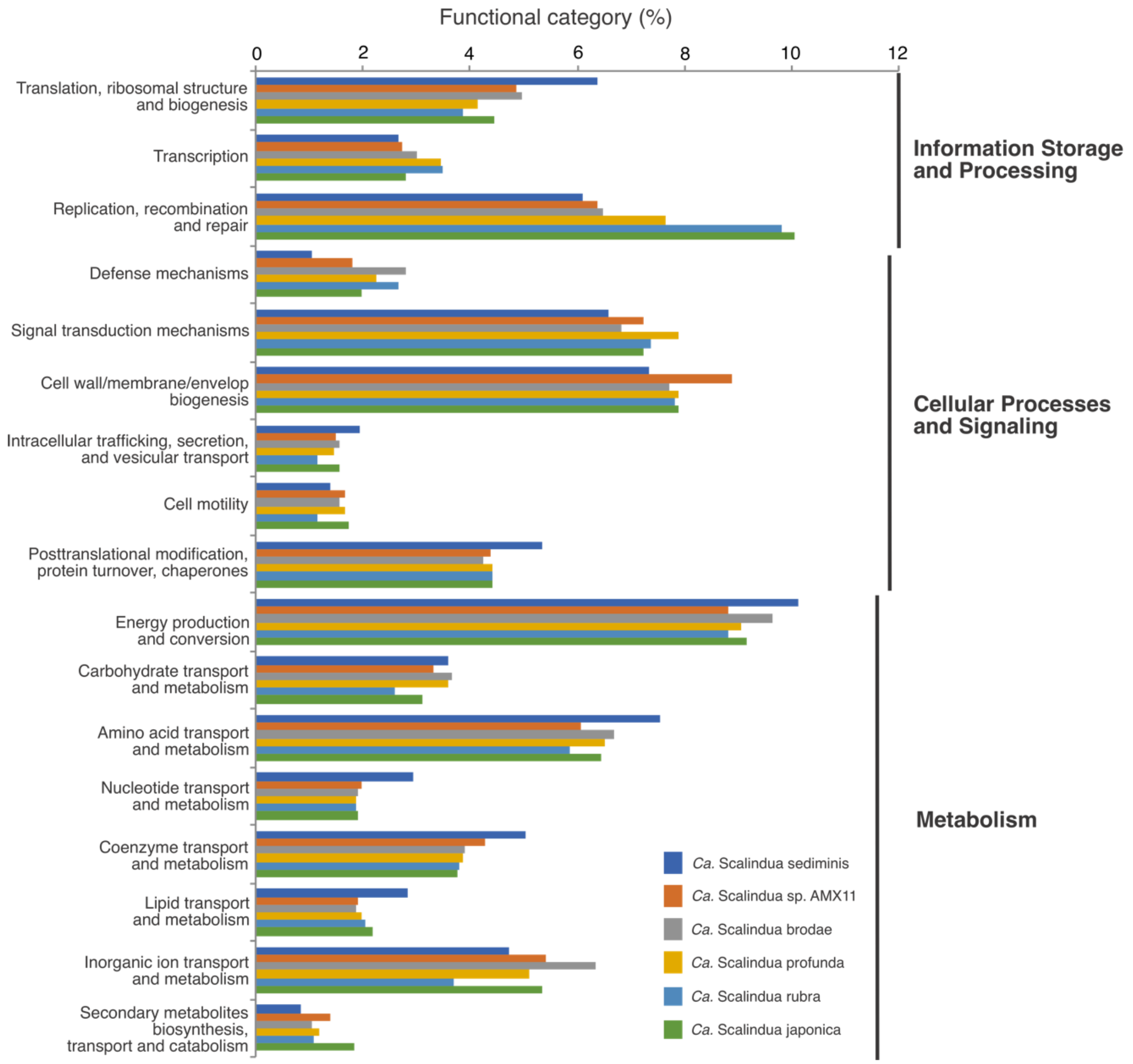

Fig. S10. Functional classification of protein-coding genes from *Ca.* Scalindua sediminis compared to other *Candidatus* Scalindua genomes. The bar chart represents the percentage of protein-coding genes classified by eggNOG functional categories. Functional categories below 1% were excluded.

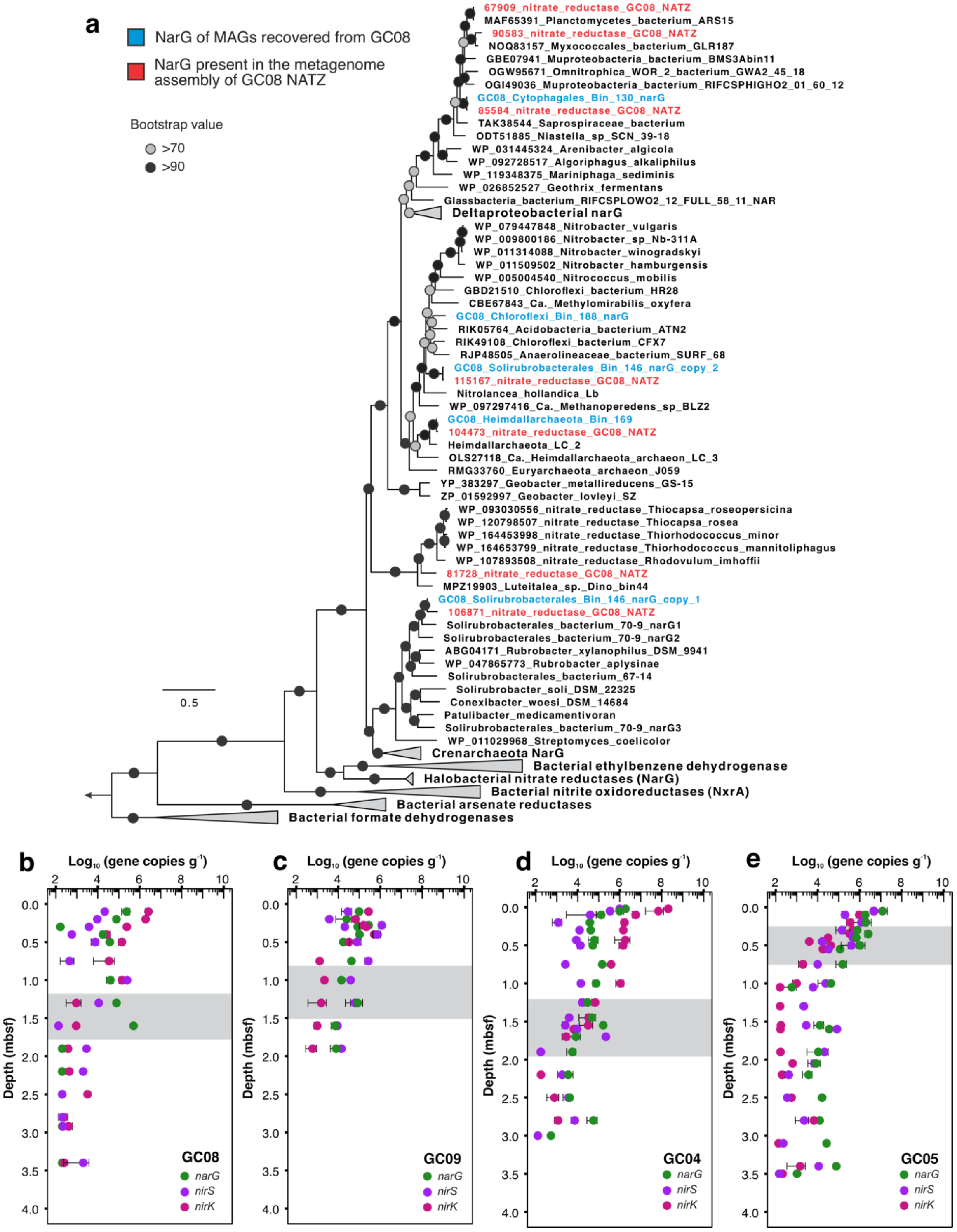

Fig. S11. Diversity and distribution of denitrifying bacteria. (a) Maximum-likelihood phylogenetic tree of periplasmic nitrate reductase alpha subunit (NarG) detected in the bulk metagenome assembly of the NATZ of GC08. Sequences from the metagenome assembly are highlighted in red, while those from the metagenome-assembled genomes (MAGs) of denitrifying bacteria recovered from this core are shown in blue. **(b-e)** q-PCR determined abundance of genes encoding nitrate reductase alpha subunit (*narG*), copper-containing nitrite reductase (*nirK*), and cytochrome *cd1*-containing nitrite reductase (*nirS*) in the four sediment cores collected from AMOR. Error bars represent standard deviations of the qPCR triplicates, and

some are smaller than the symbols and therefore are invisible. Gene abundances below detection limit were arbitrarily shown as 100 copies g^{-1}. The NATZ interval in each core is shown with a grey box.

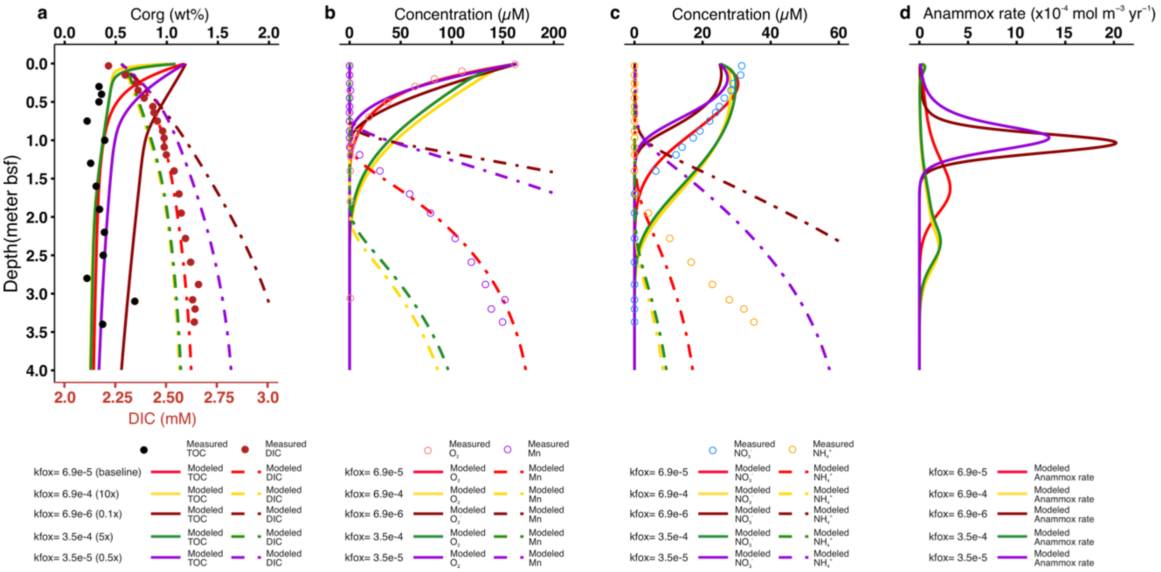

Fig. S12. Sensitivity analysis of the degradation constant (kfox) of the labile organic matter (C1). Profiles showed here are the measured (dots) and modeled concentrations (lines) of TOC and DIC **(a)**, O_2 and dissolved Mn **(b)**, NO_3^- and NH_4^+ **(c)**, and anammox rate **(d)**, with the kfox of 6.9e-5 (baseline value), 6.9e-4 (10x of the baseline value), 6.9e-6(0.1x), 3.5e-4 (5x), and 3.5e-5 (0.5x). Scales in **(a-c)** are the same as Fig. 2a-2c, while the scale of **(d)** is different from Fig. 2d.

Table S1. Properties of study sites considered in this study

Sediment core	GC08	GC09	GC04	GC05
Latitude (N)	$71^{o}97'$	$73^{o}70'$	$72^{o}16'$	$76^{o}55'$
Longitude (E)	$0^{o}10'$	$7^{o}34'$	$1^{o}42'$	$7^{o}7'$
Water depth (m)	2,476	1,653	2,668	3,007
Organic matter content (wt %)	0.3-0.6	0.2-0.5	0.3-1.0	0.3-1.8
Depth of NATZ*	1.2-1.7	0.8-1.5	1.5-2.0	0.5-0.7
Nitrate flux to NATZ**	0.27	0.24	0.22	0.64
Ammonium flux to NATZ**	0.19	0.40	0.04	0.82

*, in the unit of meters below seafloor (mbsf)
**, in the unit of mmol m^{-2} yr^{-1}

Table S2 Reaction network and rate laws

Reaction Name	Reaction	Reaction stoichiometry	Rate expression
Aerobic respiration	R_1	$\frac{1}{4}(CH_2O)(NH_3)_b + \frac{1}{4}O_2 + \frac{b}{4}H^+ \rightarrow \frac{1}{4}CO_2 + \frac{b}{4}NH_4^+ + \frac{1}{4}H_2O$	$(kfox * C_{TOC_1} + kfox_2 * C_{TOC_2}) * \frac{C_{O_2}}{C_{O_2} + h_1}$
Heterotrophic denitrification	R_2	$\frac{1}{4}(CH_2O)(NH_3)_b + \frac{1}{5}NO_3^- + \left(\frac{1}{5} + \frac{b}{4}\right)H^+ \rightarrow \frac{1}{10}N_2 + \frac{1}{4}CO_2 + \frac{b}{4}NH_4^+ + \frac{7}{20}H_2O$	$(kfox * C_{TOC_1} + kfox_2 * C_{TOC_2}) * \gamma * \frac{C_{NO_3^-}}{C_{NO_3^-} + h_2}$
Dissimilatory Mn(IV) reduction	R_3	$\frac{1}{4}(CH_2O)(NH_3)_b + \frac{1}{2}MnO_2 + \left(1 + \frac{b}{4}\right)H^+ \rightarrow \frac{1}{2}Mn^{2+} + \frac{1}{4}CO_2 + \frac{b}{4}NH_4^+ + \frac{3}{4}H_2O$	$(kfox * C_{TOC_1} + kfox_2 * C_{TOC_2}) * \gamma * \frac{h_2}{C_{NO_3^-} + h_2} * \frac{C_{MnO_2}}{C_{MnO_2} + h_3}$
Nitrification	R_4	$\frac{1}{8}NH_4^+ + \frac{1}{4}O_2 \rightarrow \frac{1}{8}NO_3^- + \frac{1}{4}H^+ + \frac{1}{8}H_2O$	$k_4 * C_{NH_4^+} * C_{O_2}$
Mn oxidation	R_5	$\frac{1}{2}Mn^{2+} + \frac{1}{4}O_2 + \frac{1}{2}H_2O \rightarrow \frac{1}{2}MnO_2 + H^+$	$k_5 * C_{Mn} * C_{O_2}$
Anammox	R_6	$\frac{1}{6}NH_4^+ + \frac{1}{6}NO_3^- + \frac{1}{3}H^+ \rightarrow \frac{1}{6}N2 + \frac{1}{2}H_2O$	$k_6 * C_{NH_4^+} * C_{NO_3^-} * \gamma$

$\gamma = \frac{h_1}{(h_1 + C_{O_2})}$, represents in the oxygen inhibition term.

Table S3. Species and boundary conditions (BC) at the sediment-water interface (SWI) used in the reaction-transport model

Name	Symbol	BC SWI Type (Unit)	BC SWI Value			
			GC08	GC09	GC04	GC05
Total organic carbon flux	CH_2O	Flux (mol m^{-2} yr^{-1})	9.30E-3	1.42E-2	1.09E-2	2.01E-2
Manganese oxide flux	MnO_2	Flux (mol m^{-2} yr^{-1})	2E-6	2.0E-5	4.0E-5	1.0E-5
Oxygen	O_2	Concentration (µM)	165	225	205	160
Ammonium	NH_4^+	Concentration (µM)	0.1	0.1	0.1	0.1
Nitrate	NO_3^-	Concentration (µM)	25	21	21	30
Manganese	Mn(II)	Concentration (µM)	0.1	0.1	0.1	0.1
DIC	HCO_3^-	Concentration (mM)	2.5	2.1	2.2	2.18

Table S4. Parameter values used in the reaction-transport model

Name	Symbol	Unit	GC08	GC09	GC04	GC05	Source	Range given by source, [Values used by source]
Sediment domain	L	cm	500	350	500	600	--	--
Solid burial velocity at compaction	ω	cm ky-1	2	5	2	2.5	a	2, [--]
TOC degradation constant C_1	kfox	1 yr^{-1}	6.9E-5	6.5E-5	6.5E-5	9.0E-5	b	1.0E-03 - 1.0E-01, [1.0E-03 - 1.0E-01]
TOC degradation constant C_2	kfox2	1 yr^{-1}	5.0E-6	2.0E-5	2.0E-6	8.0E-6	b	1.0E-06 - 7.0E-06, [1.0E-06 - 7.0E-06]
Nitrification rate constant	k_4	mM^{-1} yr^{-1}	150	150	300	150	c	5-100, [5-100]
Mn oxidation rate constant	k_5	mM^{-1} yr^{-1}	110	110	110	110	e	110, [110]
Anammox rate constant	k_6	mM^{-1} yr^{-}	50	50	50	150	f	--
Bioturbation coefficient	$D_{b,0}$	cm yr^{-1}	0	0	0	0	--	--
Biomixing half depth	z_{mix}	cm	3	3	3	3	e	5, [5]
Biomixing attenuation	Z_{att}	cm	3	3	3	3	e	5, [5]
Bioirrigation coeffcient	α_0	yr^{-1}	0	0	0	0	--	--
R_1 O_2 inhibition concentration	h_1	µM	10	10	15	10	d	1–30, [20]
R_2 NO_3^- inhibition concentration	h_2	µM	20	38	4	5	d	4-80, [2]
R_3 MnO_2 inhibition concentration	h_3	µmol g^{-1}	10	10	10	10	d	4-32, [4,32]
Porosity at sediment surface	φ_0	--	0.8	0.8	0.65	0.8	g	--
Porosity at infinite depth	φ_∞	--	0.55	0.6	0.55	0.6	g	--
Porosity attenuation coefficient	α_0	cm^{-1}	0.01	0.01	0.01	0.01	f	--

[a] Eldholm and Windisch (1974)
[b] Volz et al, (2018).
[c] Mewes et al., (2016).
[d] Wang and Van Cappellen (1996).
[e] Mogollón et al. (2016)
[f] Constrained by the model.
[g] Measured in this study.

Table S5. Root mean square error (RMSE) of porewater solutes[a]

Core ID	O_2 [μM]	Mn(II) [μM]	NO_3^- [μM]	NH_4^+ [μM]	DIC [mM]
GC08	8.1	3.7	1.8	4.6	0.03
GC09	27.2	7.9	3.1	1.2	0.07
GC04	34.8	14.3	2.3	0.5	0.07
GC05	10.0	8.0	1.1	3.4	0.08

[a] Error is calculated not for any single data point, but for the whole simulated concentration profile.

Table S6. Summary statistics of *Candidatus* Scalindua genomes

	***Ca.* S. sediminis**	***Ca.* S. rubra**	***Ca.* S. profunda**	***Ca.* S. brodae**	***Ca.* S. japonica**	***Ca.* S. AMX11**
Completeness*	95.5%	92.5%	94.2%	92.7%	95.5%	96.6%
Contamination*	4.6%	5.1%	5.4%	2.3%	3.4%	4.6%
Strain heterogeneity*	0%	0%	75%	0%	0%	25%
Total length (base pairs)	2,955,644	5,194,263	4,176,727	4,084,168	4,812,853	4,593,657
GC content	38.3%	37.3%	40.3%	39.6%	38.8%	41.1%
Number of scaffolds	71	443	3053	282	47	121
Number of contigs	83	443	4756	282	47	121
N50 of contigs	70,308	22,837	1,173	33,252	219,109	92,628
Number of coding sequences†	2,879	5,482	4,714	4,178	4,343	4095
Coding density	84.1%	80.1%	99.4%	83.9%	84.1%	81.6%
Average coverage	116.7	--	--	--	--	--

*Based on lineage-specific marker sets determined with CheckM. †Inferred with Prodigal. ‡Estimated from the proportion of reads mapped to the genome. --, No data.

Dataset S1 (separate file). Annotation of *Ca.* S. sediminis genes discussed in this study.

Dataset S2 (separate file). Measured geochemical profiles.

SI References

REFERENCES

1. *S. Arndt et al., Quantifying the degradation of organic matter in marine sediments: A review and synthesis. Earth-Science Reviews* **123***, 53-86 (2013).*

2. *P. Regnier et al., Quantitative analysis of anaerobic oxidation of methane (AOM) in marine sediments: a modeling perspective. Earth-Science Reviews* **106***, 105-130 (2011).*

3. *J. M. Mogollón, K. Mewes, S. Kasten, Quantifying manganese and nitrogen cycle coupling in manganese‐rich, organic carbon‐starved marine sediments: Examples from the Clarion‐Clipperton fracture zone. Geophysical Research Letters* **43***, 7114-7123 (2016).*

4. *K. Mewes et al., Diffusive transfer of oxygen from seamount basaltic crust into overlying sediments: An example from the Clarion–Clipperton Fracture Zone. Earth and Planetary Science Letters* **433***, 215-225 (2016).*

5. *J. B. Volz et al., Natural spatial variability of depositional conditions, biogeochemical processes and element fluxes in sediments of the eastern Clarion-Clipperton Zone, Pacific Ocean. Deep Sea Research Part I: Oceanographic Research Papers* **140***, 159-172 (2018).*

6. *R. Zhao, B. Hannisdal, J. M. Mogollon, S. L. Jørgensen, Nitrifier abundance and diversity peak at deep redox transition zones. Scientific Reports* **9***, 8633 (2019).*

7. *D. E. LaRowe et al., The fate of organic carbon in marine sediments - New insights from recent data and analysis. Earth-Science Reviews* **204***, 103146 (2020).*

8. *K. Soetaert, T. Petzoldt, Inverse modelling, sensitivity and Monte Carlo analysis in R using package FME. Journal of statistical software* **33***, 1-28 (2010).*

9. *D. C. Reed, C. K. Algar, J. A. Huber, G. J. Dick, Gene-centric approach to integrating environmental genomics and biogeochemical models. Proceedings of the National Academy of Sciences of the United States of America* **111***, 1879-1884 (2014).*

10. *J. Seeberg-Elverfeldt, M. Schlüter, T. Feseker, M. Kölling, Rhizon sampling of pore waters near the sediment/water interface of aquatic systems. Limnology and oceanography: Methods* **3***, 361-371 (2005).*

11. *L. Sororzano, Determination of ammonia in natural waters by the phenolhypochlorite method. Limnol Oceanogr* **14***, 799-801 (1969).*

12. *M. Badea et al., New electrochemical sensors for detection of nitrites and nitrates. Journal of Electroanalytical Chemistry* **509***, 66-72 (2001).*

13. *M. Stoll, K. Bakker, G. Nobbe, R. Haese, Continuous-flow analysis of dissolved inorganic carbon content in seawater. Analytical Chemistry* **73***, 4111-4116 (2001).*

14. *K. Soetaert, T. Petzoldt, F. Meysman (2010) Marelac: Tools for aquatic sciences. (R package version), pp https://cran.r-project.org/web/packages/marelac/index.html.*

15. *B. B. Jørgensen, Comparison of methods for the quantification of bacterial sulfate reduction in coastal marine sediments. II. calculation from mathematical models. Geomicrobiology Journal* **1***, 29-47 (1978).*

16. *K. Soetaert, F. Meysman, Reactive transport in aquatic ecosystems: Rapid model prototyping in the open source software R. Environmental Modelling & Software* **32***, 49-60 (2012).*

17. *K. Soetaert, T. Petzoldt, R. W. Setzer, Solving differential equations in R: package deSolve. Journal of Statistical Software* **33** *(2010).*

18. *R. C. Aller, P. O. J. Hall, P. D. Rude, J. Y. Aller, Biogeochemical heterogeneity and suboxic diagenesis in hemipelagic sediments of the Panama Basin. Deep-Sea Research Part I-Oceanographic Research Papers* **45***, 133-165 (1998).*

19. *J. W. Murray, K. M. Kuivila, Organic matter diagenesis in the northeast Pacific: transition from aerobic red clay to suboxic hemipelagic sediments. Deep-Sea Research Part a-Oceanographic Research Papers* **37***, 59-80 (1990).*

20. *S. Grandel, D. Rickert, M. Schluter, K. Wallmann, Pore-water distribution and quantification of diffusive benthic fluxes of silicic acid, nitrate and phosphate in surface sediments of the deep Arabian Sea. Deep-Sea Research Part Ii-Topical Studies in Oceanography* **47***, 2707-2734 (2000).*

21. *T. Nunoura et al., Molecular biological and isotopic biogeochemical prognoses of the nitrification-driven dynamic microbial nitrogen cycle in hadopelagic sediments. Environmental Microbiology* **15***, 3087-3107 (2013).*

22. *S. Emerson et al., Early diagenesis in sediments from the Eastern Equatorial Pacific 1: pore water nutrient and carbonate results. Earth and Planetary Science Letters* **49***, 57-80 (1980).*

23. *H. D. Schulz, A. Dahmke, U. Schinzel, K. Wallmann, M. Zabel, Early diagenetic processes, fluxes, and reaction rates in sediments of the South Atlantic. Geochimica et Cosmochimica Acta* **58***, 2041-2060 (1994).*

24. *P. N. Froelich et al., Early oxidation of organic-matter in pelagic sediments of the Eastern Equational Atlanti: suboxic diagenesis. Geochimica et Cosmochimica Acta* **43***, 1075-1090 (1979).*

25. *M. Bender et al., Organic-carbon oxidation and benthic nitrogen and silica dynamics in San Clemente Basin: a continental borderland site. Geochimica et Cosmochimica Acta* **53***, 685-697 (1989).*

26. *D. E. Canfield, B. Thamdrup, J. W. Hansen, The anaerobic degradation of organic matter in Danish coastal sediments: iron reduction, manganese reduction, and sulfate reduction. Geochimica et Cosmochimica Acta* **57***, 3867-3883 (1993).*

27. *P. Engstrom, C. R. Penton, A. H. Devol, Anaerobic ammonium oxidation in deep-sea sediments off the Washington margin. Limnology and Oceanography* **54***, 1643-1652 (2009).*

28. *A. Jaeschke et al., Molecular evidence for anaerobic ammonium-oxidizing (anammox) bacteria in continental shelf and slope sediments off northwest Africa. Limnology and Oceanography* **55***, 365-376 (2010).*

29. *M. L. Bender, K. A. Fanning, P. N. Froelich, G. R. Heath, V. Maynard, Interstitial nitrate profiles and oxidation of sedimentary organic matter in Eastern Equatorial Atlantic. Science* **198***, 605-609 (1977).*

30. *T. Freudenthal, T. Wagner, F. Wenzhofer, M. Zabel, G. Wefer, Early diagenesis of organic matter from sediments of the eastern subtropical Atlantic: Evidence from stable nitrogen and carbon isotopes. Geochimica et Cosmochimica Acta* **65***, 1795-1808 (2001).*

31. *J. P. Christensen, G. T. Rowe, Nitrification and oxygen consumption in northwest Atlantic deep-sea sediments. Journal of Marine Research* **42***, 1099-1116 (1984).*

32. *W. B. F. Ryan et al., Global multi-resolution topography synthesis. Geochemistry Geophysics Geosystems* **10***, Q03014 (2009).*

33. *J. M. Dick, Calculation of the relative metastabilities of proteins using the CHNOSZ software package. Geochemical Transactions* **9***, 10 (2008).*

34. *D. E. LaRowe, J. P. Amend, Catabolic rates, population sizes and doubling/replacement times of microorganisms in natural settings. American Journal of Science* **315***, 167-203 (2015).*

35. *L. S. Chong, M. G. Prokopenko, W. M. Berelson, A. Townsend-Small, J. McManus, Nitrogen cycling within suboxic and anoxic sediments from the continental margin of Western North America. Marine Chemistry* **128***, 13-25 (2012).*

36. *R. Barnes, K. Bertine, E. Goldberg, N2: Ar, nitrification and denitrification in southern California borderland basin sediments. Limnology and Oceanography* **20***, 962-970 (1975).*

37. *R. C. Edgar, Search and clustering orders of magnitude faster than BLAST. Bioinformatics* **26***, 2460-2461 (2010).*

38. *R. C. Edgar, UPARSE: highly accurate OTU sequences from microbial amplicon reads. Nature Methods* **10***, 996-998 (2013).*

39. *A. Lanzen et al., CREST - Classification resources for environmental sequence tags. PLoS One* **7***, e49334 (2012).*

40. *H. Wickham, ggplot2: elegant graphics for data analysis (Springer, 2016).*

41. *M. Li, Y. Hong, M. G. Klotz, J.-D. Gu, A comparison of primer sets for detecting 16S rRNA and hydrazine oxidoreductase genes of anaerobic ammonium-oxidizing bacteria in marine sediments. Applied Microbiology and Biotechnology* **86***, 781-790 (2010).*

42. *S. L. Jørgensen, R. Zhao, Microbial inventory of deeply buried cceanic crust from a young ridge flank. Frontiers in Microbiology* **7***, 820 (2016).*

43. S. F. Stoddard, B. J. Smith, R. Hein, B. R. K. Roller, T. M. Schmidt, rrnDB: improved tools for interpreting rRNA gene abundance in bacteria and archaea and a new foundation for future development. Nucleic Acids Research **43**, D593-D598 (2015).

44. H. C. Berg, The rotary motor of bacterial flagella. Annual Review of Biochemistry **72**, 19-54 (2003).

45. B. Kartal et al., Molecular mechanism of anaerobic ammonium oxidation. Nature **479**, 127-U159 (2011).

46. S. Andrews, FastQC: a quality control tool for high throughput sequence data. https://www.bioinformatics.babraham.ac.uk/projects/fastqc/ (2010).

47. A. M. Bolger, M. Lohse, B. Usadel, Trimmomatic: a flexible trimmer for Illumina sequence data. Bioinformatics **30**, 2114-2120 (2014).

48. D. H. Li, C. M. Liu, R. B. Luo, K. Sadakane, T. W. Lam, MEGAHIT: an ultra-fast single-node solution for large and complex metagenomics assembly via succinct de Bruijn graph. Bioinformatics **31**, 1674-1676 (2015).

49. Y. W. Wu, B. A. Simmons, S. W. Singer, MaxBin 2.0: an automated binning algorithm to recover genomes from multiple metagenomic datasets. Bioinformatics **32**, 605-607 (2016).

50. D. H. Parks, M. Imelfort, C. T. Skennerton, P. Hugenholtz, G. W. Tyson, CheckM: assessing the quality of microbial genomes recovered from isolates, single cells, and metagenomes. Genome Research **25**, 1043-1055 (2015).

51. B. K. Seah, H. R. Gruber-Vodicka, gbtools: interactive visualization of metagenome bins in R. Frontiers in Microbiology **6**, 1451 (2015).

52. B. Bushnell (2014) BBMap: a fast, accurate, splice-aware aligner. (Ernest Orlando Lawrence Berkeley National Laboratory, Berkeley, CA (US)).

53. S. Kumar, M. Jones, G. Koutsovoulos, M. Clarke, M. Blaxter, Blobology: exploring raw genome data for contaminants, symbionts and parasites using taxon-annotated GC-coverage plots. Frontiers in Genetics **4**, 237 (2013).

54. S. F. Altschul et al., Gapped BLAST and PSI-BLAST: a new generation of protein database search programs. Nucleic Acids Research **25**, 3389-3402 (1997).

55. T. Seemann (2015) Barrnap. Online: https://github.com/tseemann/barrnap. (Github).

56. T. Rognes, T. Flouri, B. Nichols, C. Quince, F. Mahe, VSEARCH: a versatile open source tool for metagenomics. PeerJ **4**, e2584 (2016).

57. C. Quast et al., The SILVA ribosomal RNA gene database project: improved data processing and web-based tools. Nucleic Acids Research **41**, D590-D596 (2013).

58. A. Bankevich et al., SPAdes: A new genome assembly algorithm and its applications to single-cell sequencing. Journal of Computational Biology **19**, 455-477 (2012).

59. B. K. B. Seah, H. R. Gruber-Vodicka, gbtools: Interactive Visualization of Metagenome Bins in R. Frontiers in Microbiology **6**, 1451 (2015).

60. *D. Hyatt et al., Prodigal: prokaryotic gene recognition and translation initiation site identification. BMC Bioinformatics* **11***, 119 (2010).*

61. *T. Seemann, Prokka: rapid prokaryotic genome annotation. Bioinformatics* **30***, 2068-2069 (2014).*

62. *J. Huerta-Cepas et al., eggNOG 4.5: a hierarchical orthology framework with improved functional annotations for eukaryotic, prokaryotic and viral sequences. Nucleic Acids Research* **44***, D286-D293 (2016).*

63. *M. Kanehisa, Y. Sato, K. Morishima, BlastKOALA and GhostKOALA: KEGG Tools for Functional Characterization of Genome and Metagenome Sequences. Journal of Molecular Biology* **428***, 726-731 (2016).*

64. *M. Kanehisa, S. Goto, Y. Sato, M. Furumichi, M. Tanabe, KEGG for integration and interpretation of large-scale molecular data sets. Nucleic acids research* **40***, D109-D114 (2011).*

65. *R. Sorek et al., Genome-wide experimental determination of barriers to horizontal gene transfer. Science* **318***, 1449-1452 (2007).*

66. *J. H. Campbell et al., UGA is an additional glycine codon in uncultured SR1 bacteria from the human microbiota. Proceedings of the National Academy of Sciences of the United States of America* **110***, 5540-5545 (2013).*

67. *A. M. Eren et al., Anvi'o: an advanced analysis and visualization platformfor 'omics data. PeerJ* **3***, e1319 (2015).*

68. *R. C. Edgar, MUSCLE: multiple sequence alignment with high accuracy and high throughput. Nucleic Acids Research* **32***, 1792-1797 (2004).*

69. *S. Capella-Gutierrez, J. M. Silla-Martinez, T. Gabaldon, trimAl: a tool for automated alignment trimming in large-scale phylogenetic analyses. Bioinformatics* **25***, 1972-1973 (2009).*

70. *L. T. Nguyen, H. A. Schmidt, A. von Haeseler, B. Q. Minh, IQ-TREE: A fast and effective stochastic algorithm for estimating maximum-likelihood phylogenies. Molecular Biology and Evolution* **32***, 268-274 (2015).*

71. *S. Kalyaanamoorthy, B. Q. Minh, T. K. F. Wong, A. von Haeseler, L. S. Jermiin, ModelFinder: fast model selection for accurate phylogenetic estimates. Nature Methods* **14***, 587-589 (2017).*

72. *D. T. Hoang, O. Chernomor, A. von Haeseler, B. Q. Minh, L. S. Vinh, UFBoot2: Improving the Ultrafast Bootstrap Approximation. Molecular Biology and Evolution* **35***, 518-522 (2017).*

73. *K. Katoh, D. M. Standley, MAFFT multiple sequence alignment software version 7: improvements in performance and usability. Molecular Biology and Evolution* **30***, 772-780 (2013).*

74. *H. Koch et al., Expanded metabolic versatility of ubiquitous nitrite-oxidizing bacteria from the genus Nitrospira. Proceedings of the National Academy of Sciences of the United States of America* **112***, 11371-11376 (2015).*

75. *M. Palatinszky et al., Cyanate as an energy source for nitrifiers. Nature* **524***, 105-108 (2015).*

76. *K. Kitzinger et al., Characterization of the first "Candidatus Nitrotoga" isolate reveals metabolic versatility and separate evolution of widespread nitrite-oxidizing bacteria. mBio* **9***, e01186-01118 (2018).*

77. *H. Daims et al., Complete nitrification by Nitrospira bacteria. Nature* **528***, 504-509 (2015).*

78. *D. R. Speth et al., Draft genome of Scalindua rubra, obtained from the interface above the discovery deep brine in the Red Sea, sheds light on potential salt adaptation strategies in anammox bacteria. Microbial Ecology* **74***, 1-5 (2017).*

79. *D. R. Speth et al., Draft genome sequence of anammox bacterium "Candidatus Scalindua brodae," obtained using differential coverage binning of sequencing data from two reactor enrichments. Genome Announcements* **3***, e01415-01414 (2015).*

80. *M. Oshiki et al., Genetic diversity of marine anaerobic ammonium-oxidizing bacteria as revealed by genomic and proteomic analyses of "Candidatus Scalindua japonica'. Environmental Microbiology Reports* **9***, 550-561 (2017).*

81. *M. Ali, D. R. Shaw, P. E. Saikaly, Draft genome sequence of a novel marine anaerobic ammonium-oxidizing bacterium,"Candidatus Scalindua sp.". Microbiology Resource Announcements* **8***, e00297-00219 (2019).*

82. *R. L. Tatusov, E. V. Koonin, D. J. Lipman, A genomic perspective on protein families. Science* **278***, 631-637 (1997).*

83. *I. Lagkouvardos et al., IMNGS: A comprehensive open resource of processed 16S rRNA microbial profiles for ecology and diversity studies. Scientific Reports* **6***, 33721 (2016).*

84. *M. Strous et al., Missing lithotroph identified as new planctomycete. Nature* **400***, 446-449 (1999).*

85. *T. Awata et al., Physiological characterization of an anaerobic ammonium-oxidizing bacterium belonging to the "Candidatus Scalindua" group. Appl. Environ. Microbiol.* **79***, 4145-4148 (2013).*
